# Next-Generation Sequencing of Human Mitochondrial Reference Genomes Uncovers High Heteroplasmy Frequency

**DOI:** 10.1371/journal.pcbi.1002737

**Published:** 2012-10-25

**Authors:** Maria Ximena Sosa, I. K. Ashok Sivakumar, Samantha Maragh, Vamsi Veeramachaneni, Ramesh Hariharan, Minothi Parulekar, Karin M. Fredrikson, Timothy T. Harkins, Jeffrey Lin, Andrew B. Feldman, Pramila Tata, Georg B. Ehret, Aravinda Chakravarti

**Affiliations:** 1 McKusick-Nathans Institute of Genetic Medicine, Johns Hopkins School of Medicine, Baltimore, Maryland, United States of America; 2 Johns Hopkins University Applied Physics Laboratory, Laurel, Maryland, United States of America; 3 National Institute of Standards and Technology, Gaithersburg, Maryland, United States of America; 4 Strand Life Sciences, Bangalore, India; 5 Roche Diagnostics, Indianapolis, Indiana, United States of America; 6 Life Technologies, Beverly, Massachusetts, United States of America; Cornell University, United States of America

## Abstract

We describe methods for rapid sequencing of the entire human mitochondrial genome (mtgenome), which involve long-range PCR for specific amplification of the mtgenome, pyrosequencing, quantitative mapping of sequence reads to identify sequence variants and heteroplasmy, as well as *de novo* sequence assembly. These methods have been used to study 40 publicly available HapMap samples of European (CEU) and African (YRI) ancestry to demonstrate a sequencing error rate <5.63×10^−4^, nucleotide diversity of 1.6×10^−3^ for CEU and 3.7×10^−3^ for YRI, patterns of sequence variation consistent with earlier studies, but a higher rate of heteroplasmy varying between 10% and 50%. These results demonstrate that next-generation sequencing technologies allow interrogation of the mitochondrial genome in greater depth than previously possible which may be of value in biology and medicine.

## Author Summary

This manuscript details a novel algorithm to evaluate high-throughput DNA sequence data from whole mitochondrial genomes purified from genomic DNA, which also contains multiple fragmented nuclear copies of mtgenomes (*numts*). 40 samples were selected from 2 distinct reference (HapMap) populations of African (YRI) and European (CEU) origin. While previous technologies did not allow the assessment of individual mitochondrial molecules, next-generation sequencing technology is an excellent tool for obtaining the mtgenome sequence and its heteroplasmic sites rapidly and accurately through deep coverage of the genome. The computational techniques presented optimize reference-based alignments and introduce a new *de novo* assembly method. An important contribution of our study was obtaining high accuracy of the resulting called bases that we accomplished by quantitative filtering of reads that were error prone. In addition, several sites were experimentally validated and our method has a strong correlation (R^2^ = 0.96) with the NIST standard reference sample for heteroplasmy. Overall, our findings indicate that one can now confidently genotype mtDNA variants using next-generation sequencing data and reveal low levels of heteroplasmy (>10%). Beyond enriching our understanding and pathology of certain diseases, this development could be considered as a prelude to sequence-based individualized medicine for the mtgenome.

## Introduction

The first complete human ‘genome’ sequenced was that of the mitochondrion in 1981 [1]. Since then, over 8,250 complete human and 3,220 complete non-human vertebrate mitochondrial genomes have been sequenced (http://www.ncbi.nlm.nih.gov). These contributions have come from numerous laboratories, where obtaining the complete sequence of even the ∼16.5 kb circular mitochondrial genome has been labor intensive and expensive. As an exemplar, it would be desirable to obtain this sequence on tens of thousands of samples in a simple, inexpensive, yet accurate manner. Beyond enriching many aspects of human biology, this development could be considered as a prelude, or even as a prerequisite, to sequence-based individualized medicine. Indeed, the mitochondrial genome, despite its unique structure and function, is an excellent ‘model system’ to identify and solve the technical, biological and medical problems that genomic medicine will encounter.

The mitochondrial genome (mtgenome) has multiple attractive structural and functional features. First, it is small at 16,569 bp (revised Cambridge Reference Sequence, rCRS) [2]. Second, it is divided into a small (6.8%) non-coding displacement loop (D-loop) or control region which provides the origin for mtDNA replication, and a large (93.2%) coding region compactly housing 37 genes (22 tRNAs, 13 proteins and 2 rRNAs) that encode proteins critical to the electron transport chain [1]. The unique biochemical functions of the mitochondria and its high functional content suggest that a higher fraction of mitochondrial, as compared to nuclear, mutations is likely to be functionally deleterious and have distinct phenotypes. Consequently, we have an enhanced possibility of understanding the logic of how sequence variation affects biochemical functions and organismal phenotypes. Third, depending on cell type, each cell contains hundreds or more of mitochondria, each mitochondrion harboring 2–10 genomes. Thus, the functional consequences of mtgenome variation acutely depend on the tissue, and are thus a model for all genes.

Genetic variation in the mtgenome has been critical to demonstrating its unique features of matrilineal inheritance [3], [4], lack of recombination [5], higher variability than the nuclear genome [6], [7] and hypervariability within the D-loop as compared to the rest of the mtgenome [8], [9]. These features have allowed delineation of mitochondrial haplotypes and haplogroups along maternal lines of descent in different human populations, and greatly contributed to our current understanding of human population structure and evolution. In turn, mitochondrial haplogroups have become a marker of an individual's ancestry. A surprising aspect of the mitochondrial genome has been its unusually large impact on human disease given its small size, owing to its high coding ratio and high mutation rate. The impact of mutations in the mtgenome on tissues with high-energy needs, such as muscle, has long been recognized in genetic disorders such as myoclonus epilepsy with ragged red fibers (MERRF) and Leber's hereditary optic neuropathy (LHON) [10], [11]. More broadly, mutations in the mtgenome have been identified in, or associated with, many complex disorders such as cancer, cardiovascular disease, neurodegeneration, diabetes and hearing loss [10], [12]–[16]; accumulation of mutations in the mitochondrial genome is a natural part of aging [17], [18] and the development of tumors as well [12]. Therefore, improved methods to sequence the mtgenome are of value to both biology and medicine.

The 100–1,000-fold higher mutation rate in mitochondria, as compared to the nuclear genome, is owing to the lack of a DNA repair system within the organelle [19]. Thus, alterations in the mtgenome sequence occur frequently, visualized as two or more mitochondrial genomes of different sequence within a single human. Such ‘heteroplasmy’ has long been considered rare but it is one major explanation for the variation in phenotypes between maternally related individuals with a deleterious mitochondrial mutation since different individuals within the same maternal lineage may harbor different proportions of wildtype to mutant mitochondria. However, strictly on theoretical grounds, heteroplasmy must be common since each oocyte has multiple mitochondria, as compared to the single nuclear genome. Therefore, any new mutation has a significant probability of being lost through mitochondrial segregation in the daughter cells after fertilization (mitochondrial “drift”) and needs to be balanced by additional mutations to allow variation. This may be a second reason for the higher mitochondrial mutation rate observed through heteroplasmy in all tissues. Indeed, some have proposed that, under the “mutation-drift-selection” scenario, heteroplasmy should be the default state for mtDNA in all tissues of the body from mitochondrial segregation of inherited variation or from somatic mutation [20]. Indeed, all extant mitochondrial polymorphisms must have gone through a heteroplasmic state after their origin by mutation.

A number of studies have demonstrated heteroplasmy, but its mechanism and incidence in the general population remains unknown since the detection of heteroplasmy has been hindered by the resolution of available sequencing technologies. While Sanger sequencing allows for complete coverage of the mtgenome, it is limited by the lack of deep coverage and low sensitivity for heteroplasmy detection when it is much less than 50% [21]. The Affymetrix Mitochip Array 2.0 containing the full mtDNA sense and antisense sequences tiled on an array has been successfully used in our laboratory for full mtgenome sequencing with slightly improved heteroplasmy detection [22], [23]. However, neither of these technologies allows the assessment of individual mitochondrial molecules. In contrast, next generation sequencing technology is an excellent tool for obtaining the mtgenome sequence and its heteroplasmic sites rapidly and accurately since it allows deep coverage of the genome through multiple independent sequence reads. In fact, two recent studies demonstrate that the degree of heteroplasmy can vary across an order of magnitude (typically <5% but occasionally >50%) [24] and multiple sites with the mtgenome have heteroplasmy rates >10% [25].

In this study, we present the complete mitochondrial genomic sequence and heteroplasmic status of 40 samples from the International HapMap Project [26] using the next-generation 454 GS FLX pyrosequencing platform. The samples include 20 individuals from the CEU (European ancestry) and 20 individuals from the YRI (African ancestry) reference panels; these are mtgenome sequences isolated without any contamination from nuclear embedded *numts* (see results) and from publicly available reference samples. The availability of such reference samples is critical as the samples could serve as a basis for reproducing and benchmarking new sequencing technologies. To enable analyses, we developed novel sequence processing and analysis algorithms, both for mapping against the reference sequence and for *de novo* assembly, for confident determination of the mitochondrial sequence. Our analyses demonstrate sequence accuracy of near 100%, nucleotide diversity of 1.6×10^−3^ for CEU and 3.7×10^−3^ for YRI, patterns of sequence variation consistent with earlier studies, but a high rate of heteroplasmy varying between 10% and 50%.

## Results

### Sequencing of Reference HapMap Samples

Twenty-two unique CEU (European ancestry) and twenty-two unique YRI (African ancestry) samples from the International HapMap Project [26], including two sets of duplicates for each population (CEU: NA10851, NA10856; YRI: NA18500 & NA18503), were sequenced. The DNA used was enriched for mitochondrial sequences by long range PCR (LPCR) of three ∼5–6 kb segments using mtgenome-specific primers. Although mitochondrial sequencing using total cellular DNA is possible and easy, and is being routinely performed with heteroplasmy detection [27] [28], we avoided this approach because the human nuclear genome has >1,200 non-functional mtgenome fragments (*numts*) [29] and mitochondrial pseudogenes that complicate mtgenome sequence assembly and introduces numerous polymorphism and heteroplasmic artifacts. Thus, despite its simplicity it is quite erroneous, as we will demonstrate. LPCR reduced this possibility greatly since <5% of insertion sites are >5 kb. Additionally, our primers are designed to avoid nuclear genome amplification; each primer set is specific for the mtgenome as verified by BLAST (refer to methods). We completed sequencing using the 454 GS FLX system by pooling 12 individually tagged samples into each lane of a 4-region gasket PicoTiterPlate (PTP). Two YRI samples (NA19209 and NA19116) were discarded from analysis as both samples showed only one of three amplicons with an unusually high number of sites containing two different nucleotides at high frequencies; this could have arisen from a sample mixture. In addition, two CEU samples (NA12750 and NA12872) were removed due to suspected mislabeling. The results presented are from the remaining 40 samples. On average, each sample had 10,554 reads with a standard deviation of 2,652 reads. The read length distributions were similar and consistent across all samples; the distribution across all 44 samples (including duplicates) show read lengths across a wide range but 93.7% of them are between 200–300 bp. The average read length was 250 bp with a standard deviation of 36 bp (Supporting Figure S1) so that the yield per sequencing run was ∼2.6 megabases (mb).

### Algorithm for Data Analysis

Our approach for obtaining the mtgenome sequence was to map quality filtered reads against the reference sequence (rCRS) to identify homoplasmic and heteroplasmic variant sites. We also introduce a novel method for *de novo* assembly of the reads into a circular genome. An important consideration in our study was to obtain high accuracy of the resulting called bases. We accomplished this by quantitative filtering of reads that were error prone. We finally estimated an accuracy of the resulting sequence and an analysis of its genetic features.

#### Read filtering

We used five filters that eliminated reads, which were considered either redundant (that would falsely increase accuracy) or inaccurate (that would correctly decrease accuracy). These filters built upon an earlier study to eliminate low quality data using objective criteria [30]. First, we eliminated identical (clonal) reads preserving a single copy of each set. A second filter eliminated reads containing at least one “N”. In this case, “N” is not an ambiguous base but one defined by 454 as the inability to incorporate a nucleotide after three consecutive flows in a sequencing run. Additionally, we excised the primer sequences at the beginning and ends of forward and reverse complement reads, respectively, leaving only the extended portion as part of this filter. Third, we eliminated all reads that fell outside the 200–300 bp range. A fourth filter discarded reads that did not map to rCRS or mapped to more than one location. The final filter eliminated reads that started and ended at the same position (Supporting Figure S2). These are different from clonal reads in that they were not identical in sequence, usually having a substitution or insertion/deletion (indel) within the read. Supporting Figure S3 illustrates the breakdown of reads discarded through the five filters and shows that the majority of them arose from clonal reads (∼15%), being outside the length range (∼7%) or having identical start-stops (∼9%). On average, 32% of the initial raw reads were eliminated.

#### Quantitative model for base calling

First, all quality filtered reads were aligned to rCRS (GenBank ID NC_012920), one-by-one using the BLAST algorithm [31]. Second, to identify each base at each position we focused on four parameters that affected the confidence of a call, namely, the fractional coverage for a particular base (φ), the ratio of forward-to-reverse reads for that base (ρ), the length of a homopolymeric (HP) stretch containing the mtgenome position and, the rate of substitution sequencing errors (λ). To set optimum values for each of these parameters (details below), we first compared each of the four sets of duplicate pairs sequenced, obtained their consensus sequences, and varied the parameter values until these samples showed maximum concordance across their consensus. Specific positions that did not meet the imposed threshold criteria were then denoted as ‘Z’ (un-called positions).

Specific variants inferred in the mitochondrial genome, such as those in primer sequences and homopolymeric runs of four nucleotides or more were verified manually to avoid false positives. As shown in Supporting Figure S4, the mismatch probability in homopolymeric runs, estimated from all 40 samples, increases exponentially for lengths greater than four and includes both true substitutions and sequencing anomalies. Nevertheless, homopolymeric runs are known to be highly error prone due to overcalls and/or undercalls inherent in the 454 base-calling software and alignment artifacts [32]. For judging whether reads with a secondary base at a specific position are sequence errors or heteroplasmic sites we assumed a model of random errors along the read. While the overall error rate is generally ∼0.5% [32], we assumed a more liberal value of 5% to obtain a greater confidence in heteroplasmic calls. We used this probability to calculate the expected number of substitution errors that could occur along a single read, using a Poisson approximation, so that the modal base would have 99% confidence. Positions that had secondary coverage greater than this level were then classified as potential heteroplasmic candidates for further verification. The parameter thresholds for the *primary* base call, including indels, were: φ≥0.5 (modal base), 10^6^≥ρ≥10^−6^ (at least one read in both forward and reverse directions), HP≤4 and λ = 0.05. For putative heteroplasmic sites we adopted the following thresholds for the *secondary* base call, including possible indels: φ≥0.8 (80% of leftover coverage), 12≥ρ≥1/12, HP≤4 and λ = 0.05.

#### Error and performance

To assess the quality and accuracy of the sequencing data, four sets of duplicates were sequenced: two from each population (NA10851, NA10856 from CEU; NA18500, NA18503 from YRI). Each duplicate was processed in a different region of the PicoTiterPlate (PTP). The concordance in the sequence was 100% for all sites and was based on analysis of 16,568, 16,567, 16,527 and 16,569 nucleotide positions in NA10851, NA10856, NA18500 and NA18503, respectively, or an error rate <1.51×10^−5^ (zero changes in 66,231 bp). The remaining positions were not called due to failing any of the four parameters set to call bases confidently. These four samples differed from the rCRS at 16, 30, 51 and 40 nucleotide positions, respectively. These data served as evidence to support that 454 sequencing technology together with our algorithm was robust and sufficient for accurate base calling. In addition, we compared the mtgenome sequences of four additional samples that were also sequenced using Sanger sequencing. In this analysis, the samples NA06994, NA12146, NA18516 and NA18523 showed site discordances of 0, 1, 8 and 4 nucleotide positions based on 16,567, 16,567, 16,196, 16,195 positions analyzed, respectively, an error rate of 19.84×10^−5^ (13 changes in 65,525 bp). This >10-fold difference in sequencing error demonstrates that the high coverage obtained from pyrosequencing provides very accurate data and that comparisons to Sanger sequencing create more differences due to inaccuracies in the latter. This statement was supported by comparing our results to the 1000Genomes data [27] (see below).

An additional indication of the accuracy of the sequence we generated is indicated by the frequency of the non-modal base at a given position across all samples. This provides a simple statistic that indicates how frequently an incorrect base is called since, in the majority of the samples, any given base is likely identical to the reference sequence. As shown in Supporting Figure S5, 16,462 of the 16,569 (99.4%) positions have a non-modal base frequency less than 10^−2^, indicating that a modal base can be identified at these positions without ambiguity ∼99% of the time, given the ∼120-fold level of sequence coverage.

Finally, we compared two separate *de novo* assemblies of the mtgenome (assembling the circular genome in the antegrade and retrograde directions) versus the more standard procedure of mapping reads against the rCRS, to identify potential algorithmic discrepancies for further investigation and curation. Of the 40 *de novo* assembled HapMap mtgenomes, 15 matched identically at every position when the antegrade and retrograde assemblies were compared to each other. In the other 25 cases, the ambiguities between the antegrade and retrograde assemblies were attributable to one base differences in homopolymer length calls, particularly around position 309 within the D-loop (14 cases), or to a CAFIE (carry forward and incomplete extension)-base-insertion artifact associated with long cystosine-stretches (five cases near position 309 and one case near position 16190). A consensus *de novo* assembly was obtained by resolving the homopolymer length ambiguities between forward and antegrade directions in favor of the assembly with multiplicity value, *m*>0 in both the L-strand and H-strand n-mers and then choosing the local base call with the highest local total multiplicity (see methods). This rule assumes that the 454 signal processing for homopolymer length calls gives the correct length more often than the incorrect length. Beyond a certain length, this assumption does not hold. We note that our de novo assembly for each individual are not “correct”, but do represent the compact summarizing of the 454 read data in its raw form without risking artifacts incurring mapping reads to the rCRS reference or the utilization of 454 sequence quality scores. In the general case, the ambiguities in longer homopolymers cannot be resolved without resorting to other sequencing methods with non-overlapping systematic base-call-error processes. In certain coding regions, homopolymer sequence length ambiguity may be resolvable by requiring, as an *ad hoc* rule, that the entire sequence for a protein code for a valid protein. Finally, the *de novo* assemblies were compared to those obtained by the mapping approach, and in all cases, the discrepancies were restricted to homopolymer length ambiguities as expected.

### Characteristics of mtgenome Variation

**Figure 1:**
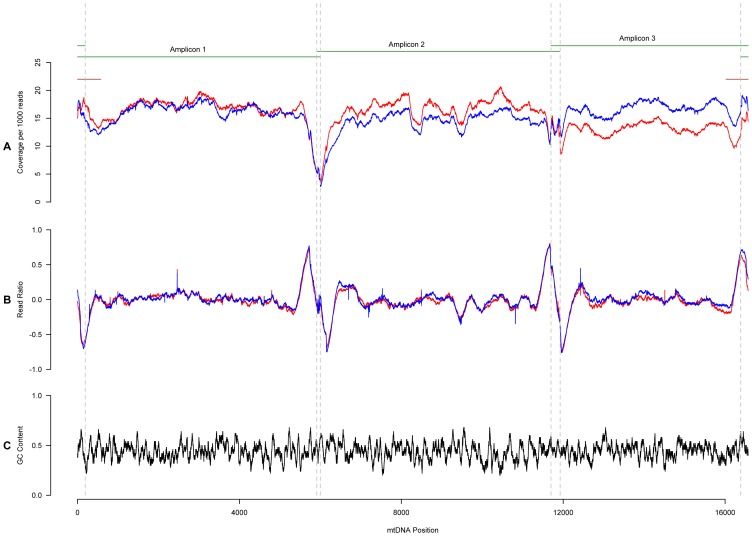
Coverage across the mitochondrial genome. The top portion of the figure shows where the three amplicons lie and overlap across the mtgenome. A) Coverage for all samples per population. For each sample the coverage at a particular position was normalized by dividing the total number of reads obtained for that sample by 1,000. B) The forward to reverse read ratio for the modal base was centered to 0 using the following statistic: [(forward/reverse)−1]/[(forward/reverse)+1]. C) GC content across the mtgenome was calculated using a sliding window of 51 bp centered on the position in question.

The overall quality of the data is summarized in Figure 1. It portrays normalized coverage and the 0-centered ratio of forward/reverse reads at each position of the mtgenome. The average coverage across all 40 samples in YRI and CEU was ∼120-fold. However, the total number of reads varied per sample so that we normalized coverage by a sample's total number of reads. Second, we assessed the directionality bias in the reads by computing ρ = (r−1)/(r+1) where *r* is the ratio of forward to reverse reads at a position. We present data on normalized coverage and read ratio as an average across the 20 samples for each population, YRI and CEU respectively. This is displayed along the mitochondrial genome (Figure 1) as a function of local GC-content, calculated using a sliding window of length 51 bp (25 bp before and after each position) across the circular genome. The figure also illustrates where the D-Loop and amplicons lie along the mitochondrial genome. As can be seen, the average coverage falls and the read ratio spikes prior to the PCR amplicon overlap regions in both populations. However, ρ fluctuations are not due to variations in GC content.

#### Frequency of polymorphic sites across the genomes

**Figure 2:**
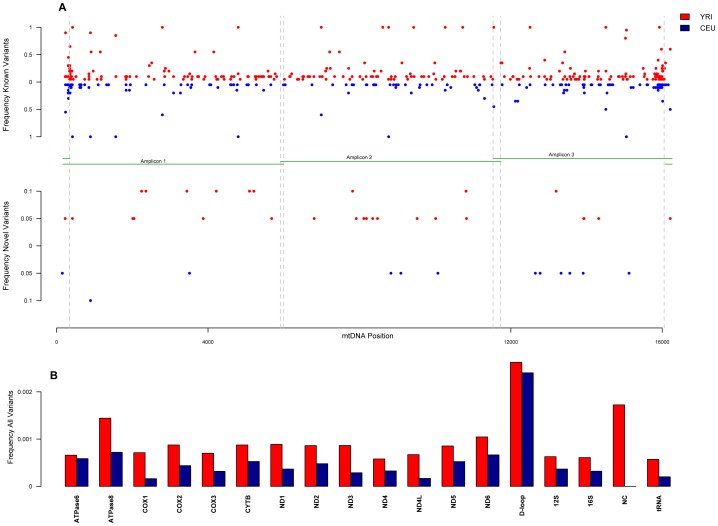
A. Frequency of polymorphic sites by population across the mitochondrial genome. The top portion of the figure shows the physical locations of the amplicons with overlap across the mtgenome; red and blue dots represent YRI and CEU samples, respectively. Frequency of known polymorphic sites for all samples by population showed on top, and frequency of novel polymorphic sites by population on bottom. The term ‘known’ refers to sites that are listed in mtDNA databases and the term ‘novel’ refers to sites that have not been previously described. **B**. **Frequency of Polymorphic sites per mitochondrial genomic region**. Red and blue bars represent YRI and CEU, respectively. Frequency was calculated by dividing the number of polymorphic sites found in each region by the length of that region and then dividing again by the sample size. Each site in a region was counted only once.

We analyzed polymorphic sites per position and per mitochondrial region as shown in Figures 2A and 2B. Figure 2A shows all the variants found for all 40 mitochondrial sequences. Variants are defined as positions where the primary base differs from the reference rCRS, and are classified by population and location across the mtgenome. Variant sites are further classified as known or novel, depending on whether or not they are recorded in the Mitomap database [7]. Overall, 418 individual variant sites (substitutions and deletions) were found across the whole mtgenome (2.6% of the entire mtgenome), including coding and non-coding regions. These sites were seen 1,069 times and 446 times in YRI and CEU, respectively. The higher frequency of variants found in YRI samples compared to CEU samples is expected due to their older population age. Figure 2A clearly shows that the majority of the variants found have already been reported in Mitomap, for both CEU and YRI. This is not unexpected since with a sample of ∼20 individuals per population the majority of recognized variants are likely to be common. Additionally, all polymorphic sites found at high frequency were previously reported in Mitomap for both populations. Novel sites were only found at low frequency; most of these were found in less than 3 individuals per population. It is evident that increased generation of sequencing data has also resulted in an increase in the number of sites reported to be polymorphic in mitomap: thus, only 28 and 12 sites had not been previously reported for YRI and CEU, respectively. Of the 418 sites, only 4 were deletions, 2 of which were common for both populations. The rest of the sites were substitutions, the majority of which were transitions (278 and 163, YRI and CEU respectively). The ratio of transitions∶transversions for all variants found, including known and novel, was 19.9:1 for YRI and 20.4:1 for CEU. This corresponds well with the values found across all populations in mtDB (19.2:1) [33], when transitions and transversions found in at least 0.2% of a sample size are considered, and by the previously reported ratio of 21.2:1 when considering only variants over the 0.1% frequency in the population [34].

The distribution of polymorphic sites per mitochondrial genomic region is shown in Figure 2B. Frequency was calculated as the number of positions found to be polymorphic for a particular region divided by the length of that region and then dividing again by the population size; positions were counted only once. As published previously, a high frequency of the polymorphic sites is found in the D-loop region as compared to other regions across the mtgenome in both populations [8], [9]. The D-loop contains 24.2% of the sites even though it is only 6.8% of the mtgenome.

To assess the accuracy of the obtained consensus sequences we compared our data to published sources [26], [27]. Concordance with HapMap II genotype data [26] was assessed for the 40 samples. For duplicates, the sequence with the highest call rate was used for comparison. HapMap II data was available for 210 SNPs. The majority of samples in the HapMap II data had a call rate higher than 98%, with the exception of five samples that had a call rate between 60–76%, giving an average call rate of 95.3±9.2% and 95.4±11.5% for CEU and YRI, respectively. The discordance rate was 0.05% for YRI and 0.4% for CEU. For the 1000Genomes genotype data [27], including that at heteroplasmic sites, information was available for 11 overlapping samples out of our 40 samples (5 CEU and 6 YRI). We compared all available genotype calls, excluding those in the error-prone homopolymeric regions where we do not provide calls, to demonstrate high concordance for all 11 samples: 8 samples were completely concordant with the remainder showing discrepancies of 2/69, 2/82, 3/69 *variant* sites. These results provide further support for the improved performance of pyrosequencing and our calling algorithm, even when compared to 1000Genomes data where *numt* contamination is a real possibility [28]. When comparing the discrepant sites obtained by Sanger chemistry for NA18516 and NA18523, 1000Genomes and our pyrosequencing data were 100% concordant. This suggests that the previously found discordances between our data and Sanger sequencing were in fact due to the inability of Sanger sequencing to pick up such variation even if high quality traces are obtained (Supporting Figure S6).

#### Heteroplasmy

Due to the resolution of next generation sequencing technologies across the entire mtgenome, it was possible to detect single nucleotide heteroplasmy even at low levels, which in our case is ∼10%. As mentioned earlier, in order to be sure that the heteroplasmic sites being detected were biologically real and not due to sequencing artifacts, we used a stringent set of criteria to call secondary bases with high confidence. Based on the chosen analysis parameters and thresholds, we were able to identify potential heteroplasmic sites. The first list of such sites was further condensed by manual curation to filter out sites that were likely the result of PCR artifacts or misalignment issues, especially at homopolymer regions. Special attention was paid to polymorphic sites within primer sequences. The latter problem arises because the reference base, the base contained in the primer, starts accumulating in the PCR product resulting in an artificial secondary base. These sites were thus regarded only as variants. Furthermore, any sites in which one of the two bases was a deletion were not taken into account. Due to alignment issues and base calling errors at homopolymeric runs discussed previously, any sites that showed two bases at the end of homopolymer stretches were also excluded.

**Figure 3:**
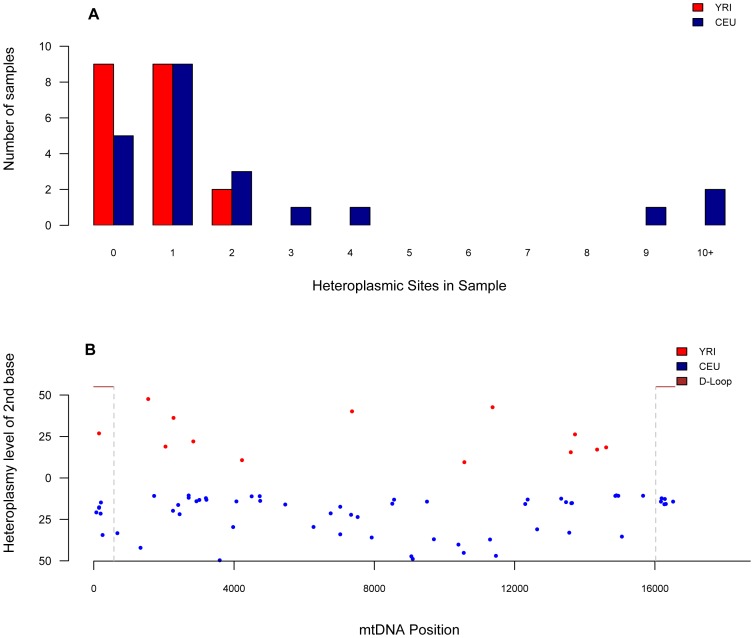
A. Heteroplasmic sites per sample. Total number of heteroplasmic sites found per sample for each YRI and CEU sample. **B**. **Level of heteroplasmy per position across the mitochondrial genome**. Top and bottom displays the level of heteroplasmy for all sites found in YRI and CEU samples, respectively, across the mitochondrial genome.

Overall, 71 sites were found to be heteroplasmic across all samples (Supporting Table S1). Curiously, CEU samples had a higher number of heteroplasmic sites per sample than the YRI samples. As shown in Figure 3A, most samples had between 0 and 3 heteroplasmic sites; only three samples showed more than 9 heteroplasmic sites, all of them in CEU. In 14 of the 40 samples, no heteroplasmic sites were identified. 58 positions were found to be heteroplasmic in at least one sample in the CEU population, two of those were seen in two different samples. YRI, on the other hand, had only 13 heteroplasmic positions, all of which were seen only once. Only one position was found to be heteroplasmic at least once in each population. As shown in Figure 3B, the detected levels of heteroplasmy ranged from 9% to ∼50% (average 22%). Figure 3B also shows the levels of heteroplasmy across the mtgenome for each population. Interestingly, heteroplasmic positions do not seem to cluster in the D-loop like other SNPs. Taken as whole, levels of heteroplasmy were higher than expected but consistent with recent studies [24], [25], except for five samples containing more than three heteroplasmic sites (Figure 3A). It is noted that while these may occur biologically, these anomalies may have arisen from two other scenarios: (1) mutations within the lymphoblastoid cell lines used as a DNA source for our experiments, or (2) inadvertent sample mixtures. Further blood samples would have to be investigated to rule out case 1. A detailed haplotype analysis was conducted for the 40 samples to rule out case (2) of mixtures within the 40 samples. Briefly, the SNPs involved did not map to different mitochondrial lineages suggesting that mixture of samples was unlikely.

Our additional analysis however supports the heteroplasmic sites we have identified. First, comparison of the four sets of duplicates showed concordance in the candidate heteroplasmic positions found, three of them having one such site (NA10851, NA10856, & NA18500). The only difference within each set was the ratio of secondary base to primary base which varied by 3%, 9% and 8% for NA10851, NA10856, & NA18500, respectively. To further check the validity of the heteroplasmic sites identified we checked the positional distribution of each heteroplasmic site along a read. The assumption driving this test was that true heteroplasmic sites should be distributed evenly across reads – at the head, in the middle and at the tail. On the other hand, false positives would be clustered either at the head or at the tail of a read because the quality of the reads decreases at the end of reads. Supporting Table S2 shows examples of true positive versus false positive sites. For most of the sites, the standard deviation of the positional distribution for the non-reference base at that site ranged from 0.6 to 6 and 0.6 to 10 in YRI and CEU, respectively. In YRI there were three outliers with standard deviations of 13, 14 and 48. CEU had 6 outliers, with standard deviations in the range 12–41.

We performed Sanger sequencing in an attempt to validate all 71 heteroplasmic sites (Supporting Table S1). 22.5% of the sites could not be validated due to poor quality of the sequencing data covering that position. Of the remaining 55 sites covered by at least one good quality read, we were able to validate 17 (29%). Of these 17, 7 were sites with low heteroplasmic levels (10%–19%). Supporting Figure S7 (A–G) shows Sanger sequence chromatograms for some of the validated sites. Surprisingly, this set also includes sites with a high level of heteroplasmy (>35%). For example, position 1333 in NA10851 did not have two bases in the chromatogram the first time we tested it. However, testing by Taqman genotyping provided evidence supporting two bases at position 1333. In a repeat experiment using Sanger sequencing we could clearly see two peaks at that position (Supporting Figure S7 B). Therefore, it is evident that we cannot rely on Sanger sequencing results for *validation* of heteroplasmic sites but need an alternative technology tested on mtgenomes purified in the same manner as ours. While the rest of the sites did not appear heteroplasmic in the chromatograms, we are confident they were true sites according to our calling parameters and the large numbers of reads supporting these calls. We also compared the status of heteroplasmy for our 11 samples that overlapped with 1000Genomes data [27]. 1000Genomes called 5 sites as heteroplasmic, 3 of which are concordant with our heteroplasmy calls. Two of them are at low levels, at 16% and 17%, while the third is at 36%. The other two sites were called only as variants by our algorithm. One of the concordant heteroplasmic sites was not validated by Sanger sequencing, which further supports the idea that Sanger sequencing is not appropriate for *validation* of heteroplasmy.

To provide further evidence supporting the accurate calling of heteroplasmy by our method, we sequenced Standard Reference Material (SRM) 2394, developed by the National Institute of Standards and Technology (NIST) [21], by both Sanger chemistry and 454 pyrosequencing. SRM 2394 is designed to simulate different levels of heteroplasmy by mixing two 285 bp mitochondrial amplicons obtained from two different human cell lines. These two amplicons differ by only one nucleotide. There are eight different mass percentages of the polymorphic mixtures, namely 1%, 2.5%, 5%, 10%, 20%, 30%, 40%, and 50%. As shown in Supporting Figure S8, there was strong correlation (R^2^ = 0.96) between our algorithm's observed heteroplasmic levels and the true values. This provides additional evidence that heteroplasmy can not only be detected but also accurately estimated at levels as low as 10%. The results of Sanger sequencing were used to visualize the different mixtures and their proportions. As expected, the chromatograms were able to resolve mixtures of 30%–50% very well; however, mixtures of 10% and 20% were barely detectable and would have been dismissed as noise if we had no previous knowledge of these mixtures.

## Discussion

The human mitochondrial genome can be sequenced at very high accuracy and rapidly using next generation sequencing technology as we, in this study, and other recent studies [24], [25], have shown. All of these studies have in common that they have uncovered patterns of sequence variation as has been described before but quantified the novel finding of a high rate of heteroplasmy in multiple individuals and across the mtgenome. Our study, however, has made three additional and important contributions. First, we have sequenced widely and publicly available biological samples so that our experiments can be replicated and provide a basis for future benchmarking and technology comparisons. Second, our methodology for variant and heteroplasmy detection is quantitative and parametric so that the method can be further optimized with additional experiments and new data. Third, we have developed a method for *de novo* sequence assembly of the mitochondrial circular genome with an internal test of sequence accuracy (identity of antegrade and retrograde assembly along a circular genome).

Each of the above developments is significant for understanding mitochondrial biology and medicine. First, DNA sequencing technology is advancing and new platforms that include single-molecule sequencing are on the horizon [35]. The availability of multiple sequencing methods on publicly available biological samples, such as those we have used, is the only certain way for comparing different technologies and their relative advantages and disadvantages. Second, we believe that the parameters we have used for identifying variants and heteroplasmy will need to be varied depending on the specific technology used and its features such as directional bias, read accuracy, difficulty in reading through homopolymeric tracts and coverage. Consequently, our approach is general and generalizable. Third, mapping reads against a reference suffers from the disadvantage of not being able to confidently identify insertions or inversions. The *de novo* methods we have introduced can rectify this deficiency particularly since our preliminary exploration of 40 sequences suggests that it produces high-quality assemblies.

The problems associated with recovery of target mitochondrial DNA from a biological sample, its DNA sequencing using short reads, the assembly of these reads into an mtgenome and its interpretation of variation and heteroplasmy are invariably confounded. We chose to recover the mtgenome in each individual by three distinct long-range PCR segments, analogous to Li et al. (2010) and in contrast to He et al. (2010). Our primers are designed to specifically target mtDNA and avoid introducing any artifacts from the numerous mitochondrial fragments (*numts*) in the nuclear human genome. Even if there is indeed some contamination from *numts*, this effect is expected to be small since it is assumed that there are many more copies of the entire mtgenome than two *numts* copies per the >1,200 autosomal insertion sites. However, specific fragments are present in >100 copies and can, and do, get amplified [29]. We expect that single molecule sequencing will reduce or eliminate this potential technical artifact. It is currently popular to extract and assemble the mitochondrial genome from whole genome sequencing of total cellular DNA [27]; Picardi and Pesole (2012) have recently done so from off-target exome sequencing data. But, these latter authors also show that ∼1% of all reads map to the mtgenome and not to known *numts*! Consequently, extensive filtering may be necessary to derive the mtgenome but this might also lose the genome-specific features including heteroplasmic sites. In other words, comparison of our data with those of others needs to consider how the mt DNA was isolated in the first place.

In this study, we have made no attempt to estimate the cost of sequencing a single mtgenome in any accurate way. In any case, we have demonstrated that we can obtain such sequence rapidly and with an error rate <5.63×10^−4^. Our crude estimate is that each sequence can be obtained for ∼$50 at high throughput much of this cost being the cost of mt DNA recovery. If so, studies of an entire cohort of individuals who have been measured for numerous medically relevant traits and are being followed for disease outcomes would be an ideal pilot experiment for individualized medicine.

## Materials and Methods

### DNA Samples

Forty-four reference DNA samples of unrelated individuals from the International HapMap project were studied using 454 pyrosequencing technology. The samples included 22 Yoruba samples from Nigeria (YRI: NA18500, NA18503, NA18506, NA18516, NA18523, NA18852, NA18855, NA18858, NA18861, NA18870, NA18912, NA19092, NA19101, NA19116, NA19137, NA19140, NA19152, NA19159, NA19171, NA19200, NA19203 & NA19209) and 22 Utah residents of European ancestry (CEU: NA06993, NA06994, NA07019, NA10851, NA10854, NA10856, NA10863, NA11831, NA11881, NA11882, NA11995, NA12004, NA12005, NA12144, NA12145, NA12146, NA12156, NA12248, NA12750, NA12760, NA12872 & NA12891), four of which were studied in duplicate (NA18500 and NA18503 from YRI; NA10851 and NA10856 from CEU). Additionally, four of these samples were sequenced using Sanger sequencing and the Affymetrix Mitochip Array 2.0 (NA06994, NA12146, NA18516, and NA18523) for comparison. We also evaluated the Standard Reference Material (SRM) 2394 developed by the National Institute of Standards and Technology (NIST). These are a set of eight mixtures (mass percentages of 1%, 2.5%, 5%, 10%, 20%, 30%, 40%, and 50%) of two 285 bp mitochondrial amplicons that differ in sequence by only one nucleotide and is obtained from two different human cell lines. After QC checks that detected sample contamination, data from NA19209, NA19116, NA12750 and NA12872 were dropped from further analysis.

### Sample Preparation

For pyrosequencing, we enriched for the mitochondrial genomic DNA by long range PCR (∼5–6 Kb) for three overlapping amplicons using high-fidelity TaKaRa LA *Taq* (TaKaRa Biomedicals) in 50 µl reactions (50 ng gDNA, 1× LA PCR buffer, 0.3 µM of each primer, 400 µM dNTPs, 2.5 U LA Taq). The primer sequences used were those described in Maitra et al (2004). Each primer set was blasted against the entire human genome to verify that there was no nuclear genome amplification. In silico PCR also confirmed no nuclear genome targets amplification by any of the three distinct primer sets. The success of the amplification reaction was checked by gel electrophoresis. The PCR products were then cleaned using the QIAquick PCR purification kit (QIAGEN) following the column purification protocol and the DNA was eluted in 30 µl of Elution Buffer to obtain a higher concentration. The actual concentration was determined using the Quant-iT PicoGreen dsDNA kit (Invitrogen). To obtain a uniform representation of the entire mtgenome, the amplicons were pooled in equimolar amounts (amount per amplicon [ng] = fraction of total x total amount needed). Since the pyrosequencing protocol required more than 5 µg of total DNA at a concentration of 300 ng/µl we performed at least two PCR reactions per amplicon. After pooling the three amplicons per reference sample in equimolar amounts, the samples were run through a QIAquick purification column to concentrate the pool to the desired 300 ng/µl concentration.

For Sanger sequencing, the mtgenome was amplified in 24 overlapping PCR fragments (800–900 bp) as described in Rieder et al 1998. For easy detection during sequencing, M13 tags were added to all forward and reverse primer sets. PCR reactions and cycling conditions were optimized across all primer sets and used 1× PCR Buffer, 200 µM dNTP, 0.5 U Taq2000, 10 ng DNA, and 0.5 µM of each primer. Confirmation of the reactions' specificity was assessed by 2% agarose gel electrophoresis. The final concentration of each amplicon was determined using the Quant-iT PicoGreen dsDNA kit (Invitrogen).

### DNA Sequencing and Primary Analysis

All sequencing using Sanger chemistry were performed by a commercial entity (Agencourt) for each individual PCR product on an automated ABI3730xl platform using a concentration of 15–25 ng/µl in 30 µl of TE buffer; individual sequence traces were provided. The Sanger sequence for each sample was assembled and analyzed in the SeqManII program from the DNASTAR Lasergene® v.7.0 analysis software suite. All sequencing reads for an individual sample were imported and assembled into one contiguous consensus sequence by aligning them to the revised Cambridge Reference Mitochondrial Sequence (rCRS). The variant bases for each sample were determined and used as the genotype for that sample for further analysis. Peak intensities for each sequence variant identified by the program were manually reviewed.

For pyrosequencing of the 48 samples, including duplicates, we pooled the pooled long range PCR products per sample in four batches of 12 each using 454's Multiplex Identifiers (MID) that are molecular barcodes that serve as unique tags to identify each sample post-sequencing. These mitochondrial DNA pools were sequenced on a 4-gasket PicoTiterPlate (PTP) using the GS FLX sequencing system. Standard emPCR and sample preparation were followed as recommended by the manufacturer (Roche Inc.)

### Modified BLAST to Improve Alignment

As an additional precaution against misalignments, we developed an improved version of the BLAST algorithm. BLASTN uses an affine gap costs model and allows control of gap opening, gap extension and mismatch penalties and are particularly problematic for homopolymer stretches due to undercalls and overcalls. To accurately align these reads against a reference sequence, we needed an aligner that adjusts the gap penalties depending on the presence and length of the homopolymer sequence. The standard Smith-Waterman algorithm for aligning two sequences can be extended to handle these situations as follows. Let c(n,m) be the penalty for a n-length homopolymeric stretch of the reference appearing as an m-length stretch in the read. Then, the dynamic programming algorithm was modified to consult the c matrix also when computing the optimal alignment of the sequences. The entries of the c(n,m) matrix needed to be defined heuristically. In the current study, we set c(n,m) such that in homopolymer stretches of length ≥5, two gaps were ignored and the remaining penalized using the standard affine gap penalty model of BLASTN. In homopolymeric stretches of length 4, one gap was ignored. Since the largest homopolymeric stretch in the mitochondrial sequence is only 8 bases long, these values in the c(n,m) table were sufficient to yield good results. For performance reasons, we carried out alignment first using BLAST. Portions of the resultant alignment that were likely to benefit from our homopolymer-aware aligner were identified and refined using a Perl implementation of the model described above.

### *De novo* Assembly of the Mitochondrial Genome

We developed an independent *de novo* assembly of each mtgenome. In our approach, we initially populate a database comprising all unique n-mers (*n* = 27 here) and the frequency of each n-mer in the raw read data. To populate the database we slide a window, *n* bases long, along each read and record the sequence within the window as the read is traversed. Starting at the first base position, the n-mer comprising the first base and the subsequent n-1 bases is recorded. The window position is then incremented 1 base at a time until all n-mers from the read have been entered into the database. If an n-mer sequence already exists in the database, the number of occurrences (multiplicity, *m*) is incremented by 1. As an example, the distribution of *m* over all 454 reads for sample NA06993 is shown in Supporting Figure S9. The distribution is multimodal. The peak at multiplicity *m* = 1 comprises all n-mers that contain one or more 454 sequencing errors and that are not repeated as a group in any other read of the particular region of the genome. The peak near *m* = 50 is the mode of the local, n-mer matched, consensus coverage of the genome. The high multiplicities in the tail of the distribution are due to genomic regions where the long PCR segments overlap.

To *de novo* assemble the mtgenome using the n-mer database data, we make the following minimal set of assumptions: 1) there are no duplicated n-mers within the genome; 2) there are no palindromic n-mers, i.e., an n-mer on the L-strand of the mtGenome is not found in reverse complement form on the H-strand and vice versa, and 3) for a short n-mer drawn from the genome, the sequence read of this n-mer is more likely to be correct than contain an error. The third assumption depends on the sequence-context-dependent error rate of the 454 platform. If we consider as a characteristic value, λ = 0.005, for the average 454 error rate per base, then for any n-mer, the probability that the n-mer is error free is given by p = (1−λ)^n^. If we choose *n* = 27, this gives *p* = 0.87. This means that a majority of database n-mers are correct given that most of the mtgenome sequences are “average in content”. This calculation assumes errors are uncorrelated along the n-mer, which is *not the case for the sequence context of long homopolymeric runs* (see below). Our choice of n = 27 is a compromise value that seeks to ensure the validity of assumptions 1–3: the shorter an n-mer is, the more likely it is to be repeated in the mtgenome or be a palindrome; on the other hand, if the n-mer is chosen to be too long, the majority of n-mers derived from reads at a given genome position will contain an error somewhere within the n-mer. The satisfaction of assumption 3 allows us to apply a “majority base wins” criterion as our basis for selecting sequences in our *de novo* consensus assembly.

The *de novo* assembly initially proceeds by searching the database for the n-mer matching at position 1 on the L-strand of the rCRS and ensuring the multiplicity *m* for L- strand and H- strand sequences at this position exceed 10. This starting condition was satisfied for all mtgenomes assembled (i.e., no genome contained a polymorphism with respect to the rCRS in this portion of the genome, otherwise successive positions along the rCRS could readily be probed until this condition was satisfied). First, *de novo* assembly proceeds in the antegrade direction (with increasing rCRS position). We form the four possible candidates for the successive n-mer in the sequence and their respective reverse complements by dropping the first base of the n-mer at rCRS position 1 and adding A, T, C, or G to the end. The database is then searched for each candidate n-mer and its reverse complement, and the sum of the respective forward and reverse n-mer multiplicities is recorded for each candidate n-mer. According to assumption 3), the appropriate choice of the subsequent n-mer is the one that is the most abundant in the database. The selected new base is then added to the *de novo* assembly and the process is repeated until the starting n-mer sequence at rCRS position 1 is again encountered (exploiting the circular nature of the mtgenome). The antegrade *de novo* assembly is then complete. To assign consensus coverage at each base position we form the n-mer from the antegrade assembly in which the position in question is at the center, with (n_mer-1)/2 bases on either side. The database is then searched for this n-mer and the sum of the L-strand and H- strand multiplicites, *m*, is recorded as the consensus coverage.

As a check on the antegrade *de novo* consensus assembly, the entire assembly process above is repeated by sequencing from rCRS position 1 in the retrograde direction using the same database. Here, the base at the end of the L-strand n-mer is dropped and the candidate n-mers for the next position in the retrograde direction are formed by adding A, T, C, or G to the beginning of the n-mer. The alternative assemblies in the antegrade and retrograde directions are subsequently compared to identify discrepancies for further investigation and curation.

Substitution heteroplasmy candidates, and their respective fractions with respect to the consensus sequence, can then be determined by replacing the central base at each position with the other three possible bases, and then summing the L-side and H-side multiplicities of the n-mers in the database. Indel heteroplasmys with respect to the consensus can also be determined using a method aligning the unused n-mers in the database against the consensus.

## Supporting Information

Figure S1 **Average read length distribution for 40 samples including duplicates.** After removing clonal reads, the average read length was 249.9 nt with a standard deviation of 36 nt; 93.7% of reads were between 200 and 300 nt long.
(TIFF)

Figure S2 **Example of reads located in primer overlap between amplicons 2 and 3 for sample NA18870.** A) All reads spanning the overlap region of amplicons 2 and 3. B) Reads remaining after eliminating those that start and end at the same position. The discarded reads are not identical to each other.
(TIFF)

Figure S3 **Quality control (QC) filters.** Reads were filtered using five different criteria to retain only high quality reads by removing: 1. clonal reads; 2. reads containing at least one N (N does not indicate an ambiguous base but is defined as an instance when a nucleotide was not incorporated after three consecutive flows in a sequencing run); 3. reads longer than 300 or shorter than 200 nt based on the read length distribution observed across all samples; 4. reads mapping to multiple locations or not mapping to the mitochondrial genome; and, 5. reads of equal length starting and ending at the same positions but not identical to each other. On average 68% and 66% were preserved for CEU and YRI, respectively.
(TIFF)

Figure S4 **Sequence error as function of homopolymeric region.** Homopolymer stretches were grouped by length and the total number of non-consensus bases within each stretch was divided by the total number of bases in that group. The probability of having a substitution or mismatch (majority are deletions) exponentially increases in homopolymer regions of length greater than 4 nt. Final positions in such regions were not called due to this intrinsic error in the 454 pyrosequencing base-calling algorithm along with the BLAST alignment.
(TIFF)

Figure S5 **Fraction of total non-consensus bases.** The fraction of total non-consensus bases was calculated by dividing the total number of non-consensus bases at a particular position by the total number of bases seen at that position. The results indicate that 99% of all positions have secondary coverage fraction less than 1.7×10^−2^, 2.1×10^−2^ for YRI, CEU respectively.
(TIFF)

Figure S6 **Sanger sequencing chromatograms for sample NA18516.** The traces show the region containing 8 discrepancies between pyrosequencing and Sanger sequencing data. The pyrosequencing calls are also supported by 1000Genomes data. The whole region is covered by high quality bases.
(TIFF)

Figure S7 **Sanger sequencing chromatograms showing heteroplasmic sites.** Sanger sequence traces showing validation of both lower (10%–19%) and higher (>20%) level heteroplasmic sites. A) Bases around position 13328 for sample NA07019. Position 13328, next to the blue line, has two bases, namely a C and a T. The level of heteroplsmy for this site was calculated as 13% in our analysis. B) Position 1333 in sample NA10851 has two bases, G and A at almost 50%, close to the 42% in our analysis. C) Position 7925 in sample NA10863 next to the blue line, has two bases, G and A; calculated at 36% D) Position 251 in sample NA12145 next to blue line has two bases, G and A; calculated at 34%. E) Position 8512, next to blue line, shows a lower level heteroplasmic site (16%), A and G. F) Two traces covering the region around position 2274, show A and G heteroplasmy at that site, calculated as 36%. G) Position 7364 in sample NA19152 showing both A and G, calculated at 40%, and H) Position 1552 in sample NA19203 showing G and A, calculated at 48%.
(TIFF)

Figure S8 **Accuracy of heteroplasmy detection.** The Standard Material (SRM 2394) is designed to simulate different levels of heteroplasmy by mixing two 285 bp amplicons obtained from two different cell lines which differ by one nucleotide in the following mass percentages: 1%, 2.5%, 5%, 10%, 20%, 30%, 40%, and 50%. The graph shows the strong relationship between the true versus estimated mass percentage with coefficient of determination R^2^ = 0.96.
(TIFF)

Figure S9 **n-mer multiplicity.** Frequency of occurrence (multiplicity) of n-mers for n = 27 obtained from 454 reads from a single HapMap sample (NA06993).
(TIFF)

Table S1 **Heteroplasmic sites and validation status by sample.** The table provides for each heteroplasmic site its position, the relevant bases, coverage, heteroplasmic frequency, validation status and whether it was also identified in 1000Genomes. A) Heteroplasmic sites found in YRI samples and B) CEU samples.
(DOCX)

Table S2 **Positional distribution of a particular heteroplasmic site along a read.** The symbol c( ) is used to count the number of times the primary base at any position is found at the beginning, middle or end of a read, and d( ) is used in the same way for counts of the secondary base at that position. A) Two strong heteroplasmic candidate sites showing a uniform positional distribution. B) Three artifactual heteroplasmic candidate sites displaying a biased distribution along the read. These sites are all contained in primer sequences.
(DOCX)

## References

[1] Anderson S, Bankier AT, Barrell BG, de Bruijn MHL, Coulson AR, et al (1981) Sequence and organization of the human mitochondrial genome. Nature 290: 457–465. 7219534 10.1038/290457a0

[2] Andrews RM, Kubacka I, Chinnery PF, Lightowlers RN, Turnbull DM, et al (1999) Reanalysis and revision of the cambridge reference sequence for human mitochondrial DNA. Nat Genet 23: 147. 10508508 10.1038/13779

[3] Hutchinson CA, Newbold JE, Potter SS, Edgell MH (1974) Maternal inheritance of mammalian mitochondrial DNA. Nature 251: 536–538. 4423884 10.1038/251536a0

[4] Giles RE, Blanc H, Cann HM, Wallace DC (1980) Maternal inheritance of human mitochondrial DNA. Proc Natl Acad Sci U S A 77: 6715–6719. 6256757 10.1073/pnas.77.11.6715 PMC350359

[5] Olivo PD, Van de Walle, Michale J, Laipist PJ, Hauswirth WW (1983) Nucleotide sequence evidence for rapid genotypic shifts in the bovine mitochondrial DNA D-loop. Nature 306: 400. 6646218 10.1038/306400a0

[6] Brandon MC, Lott MT, Nguyen KC, Spolim S, Navathe SB, et al (2005) MITOMAP: A human mitochondrial genome database–2004 update. Nucleic Acids Res 33: D611–613. 15608272 10.1093/nar/gki079 PMC540033

[7] Ruiz-Pesini E, Lott MT, Procaccio V, Poole JC, Brandon MC, et al (2007) An enhanced MITOMAP with a global mtDNA mutational phylogeny. Nucleic Acids Res 35: D823–D828. 17178747 10.1093/nar/gkl927 PMC1781213

[8] Upholt WB, Dawid IB (1977) Mapping of mitochondrial DNA of individual sheep and goats: Rapid evolution in the D loop region. Cell 11: 571–583. 884736 10.1016/0092-8674(77)90075-7

[9] Aquadro CF, Greenberg BD (1983) Human mitochondrial DNA variation and evolution: Analysis of nucleotide sequences from seven individuals. Genetics 103: 287–312. 6299878 10.1093/genetics/103.2.287 PMC1219980

[10] Wallace DC, Zheng X, Lott MT, Shoffner JM, Hodge JA, et al (1988) Familial mitochondrial encephalomyopathy (MERRF): Genetic, pathophysiological, and biochemical characterization of a mitochondrial DNA disease. Cell 55: 601–610. 3180221 10.1016/0092-8674(88)90218-8

[11] Newman NJ, Lott MT, Wallace DC (1991) The clinical characteristics of pedigrees of leber's hereditary optic neuropathy with the 11778 mutation. Am J Ophtalmol 111: 750. 10.1016/s0002-9394(14)76784-4 2039048

[12] Warburg O (1956) On the origin of cancer cells. Science 123: 309–314. 13298683 10.1126/science.123.3191.309

[13] Holt IJ, Harding AE, Petty RK, Morgan-Hughes JA (1990) A new mitochondrial disease associated with mitochondrial DNA heteroplasmy. Am J Hum Genet 46: 428. 2137962 PMC1683641

[14] Kadowaki T, Kadowaki H, Mori Y, Tobe K, Sakuta R, et al (1994) A subtype of diabetes mellitus associated with a mutation of mitochondrial DNA. N Engl J Med 330: 962–968. 8121460 10.1056/NEJM199404073301403

[15] Gerbitz KD, Gempel K, Brdiczka D (1996) Mitochondria and diabetes. genetic, biochemical, and clinical implications of the cellular energy circuit. Diabetes 45: 113–126. 8549853 10.2337/diab.45.2.113

[16] Schon E, Bonilla E, DiMauro S (1997) Mitochondrial DNA mutations and pathogenesis. J Bioenerg Biomembr 29: 131–149. 9239539 10.1023/a:1022685929755

[17] Wallace DC (1992) Mitochondrial genetics: A paradigm for aging and degenerative diseases? Science 256: 628–632. 1533953 10.1126/science.1533953

[18] Wallace DC. (2001; 2008) A mitochondrial paradigm for degenerative diseases and ageing. In: Anonymous Ageing Vulnerability: Causes and Interventions. John Wiley & Sons, Ltd. pp. 247–266. 10.1002/0470868694.ch20 11280029

[19] Bogenhagen DF (1999) Repair of mtDNA in vertebrates. Am J Hum Genet 64: 1276–1281. 10205257 10.1086/302392 PMC1377862

[20] White DJ, Wolff JN, Pierson M, Gemmell NJ (2008) Revealing the hidden complexities of mtDNA inheritance. Mol Ecol 17: 4925–4942. 19120984 10.1111/j.1365-294X.2008.03982.x

[21] Hancock DK, Tully LA, Levin BC (2005) A standard reference material to determine the sensitivity of techniques for detecting low-frequency mutations, SNPs, and heteroplasmies in mitochondrial DNA. Genomics 86: 446–461. 16024219 10.1016/j.ygeno.2005.06.006

[22] Maitra A, Cohen Y, Gillespie SED, Mambo E, Fukushima N, et al (2004) The human MitoChip: A high-throughput sequencing microarray for mitochondrial mutation detection. Genome Res 14: 812–819. 15123581 10.1101/gr.2228504 PMC479107

[23] Hartmann A, Thieme M, Nanduri LK, Stempfl T, Moehle C, et al (2009) Validation of microarray-based resequencing of 93 worldwide mitochondrial genomes. Hum Mutat 30: 115–122. 18623076 10.1002/humu.20816

[24] He Y, Wu J, Dressman DC, Iacobuzio-Donahue C, Markowitz SD, et al (2010) Heteroplasmic mitochondrial DNA mutations in normal and tumour cells. Nature 464: 610–614. 20200521 10.1038/nature08802 PMC3176451

[25] Li M, Schönberg A, Schaefer M, Schroeder R, Nasidze I, et al (2010) Detecting heteroplasmy from high-throughput sequencing of complete human mitochondrial DNA genomes. Am J Hum Genet 87: 237–249. 20696290 10.1016/j.ajhg.2010.07.014 PMC2917713

[26] International HapMap Consortium (2003) The international HapMap project. Nature 426: 789–796. 14685227 10.1038/nature02168

[27] Altshuler DL, Durbin RM, Abecasis GR, Bentley DR, Chakravarti A, et al (2010) A map of human genome variation from population-scale sequencing. Nature 467: 1061–1073. 20981092 10.1038/nature09534 PMC3042601

[28] Picardi E, Pesole G (2012) Mitochondrial genomes gleaned from human whole-exome sequencing. Nat Meth 9: 523–524. 10.1038/nmeth.2029 22669646

[29] Gherman A, Chen PE, Teslovich TM, Stankiewicz P, Withers M, et al (2007) Population bottlenecks as a potential major shaping force of human genome architecture. PLoS Genet 3: e119. 17658953 10.1371/journal.pgen.0030119 PMC1925129

[30] Huse SM, Huber JE, Morrison HG, Sogin ML, Welch DM (2007) Accuracy and quality of massively parallel DNA pyrosequencing. Genome Biol 8: R143. 17659080 10.1186/gb-2007-8-7-r143 PMC2323236

[31] Altschul SF, Gish W, Miller W, Myers EW, Lipman DJ (1990) Basic local alignment search tool. J Mol Biol 215: 403–410. 2231712 10.1016/S0022-2836(05)80360-2

[32] Quinlan AR, Stewart DA, Stromberg MP, Marth GT (2008) Pyrobayes: An improved base caller for SNP discovery in pyrosequences. Nat Methods 5: 179–181. 18193056 10.1038/nmeth.1172

[33] Ingman M, Gyllensten U (2006) mtDB: Human mitochondrial genome database, a resource for population genetics and medical sciences. Nucleic Acids Res 34: D749–D751. 16381973 10.1093/nar/gkj010 PMC1347373

[34] Pereira L, Freitas F, Fernandes V, Pereira JB, Costa MD, et al (2009) The diversity present in 5140 human mitochondrial genomes. Am J Hum Genet 84: 628–640. 19426953 10.1016/j.ajhg.2009.04.013 PMC2681004

[35] Sam LT, Lipson D, Raz T, Cao X, Thompson J, et al (2011) A comparison of single molecule and amplification based sequencing of cancer transcriptomes. PLoS ONE 6: e17305. 21390249 10.1371/journal.pone.0017305 PMC3046973

