# Supplementary figures and images for: Next-Generation Sequencing of Human Mitochondrial Reference Genomes Uncovers High Heteroplasmy Frequency
Source: PLoS Comput Biol. 2012 Oct 25;8(10):e1002737. doi: 10.1371/journal.pcbi.1002737 (PMC3486893; doi:10.1371/journal.pcbi.1002737)

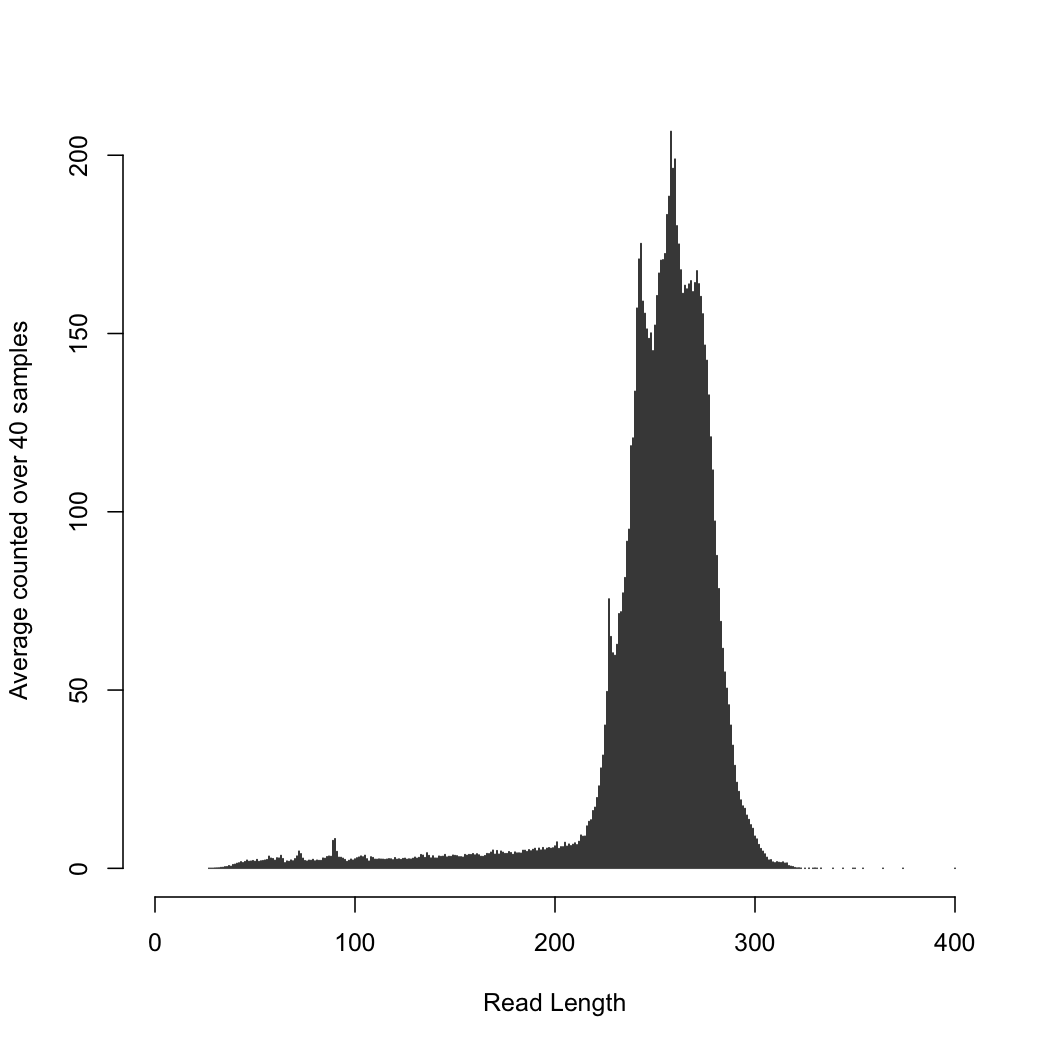

Supplement: Figure S1 — Average read length distribution for 40 samples including duplicates. After removing clonal reads, the average read length was 249.9 nt with a standard deviation of 36 nt; 93.7% of reads were between 200 and 300 nt long. (TIFF) [file pcbi.1002737.s001.tif]

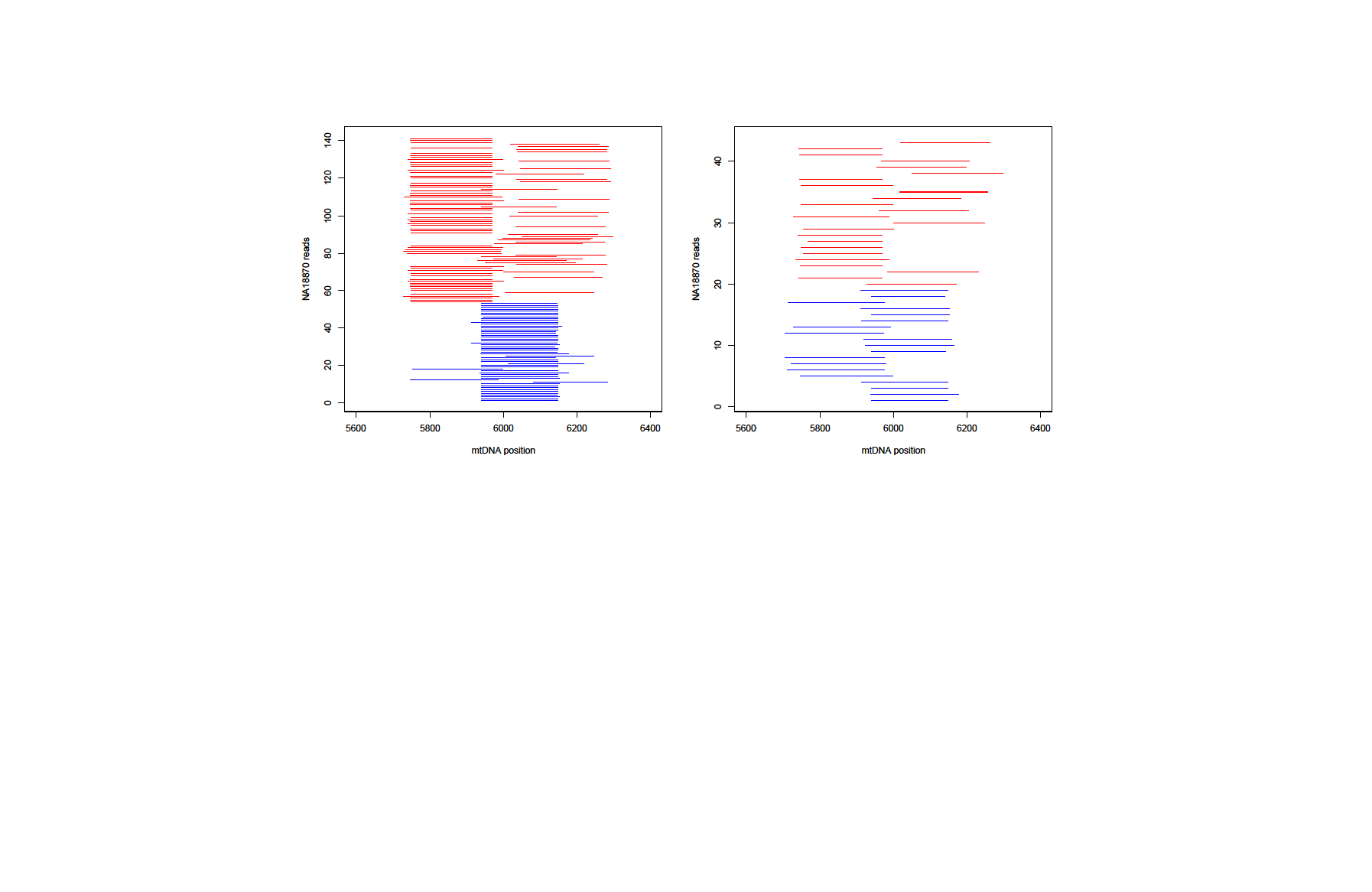

Supplement: Figure S2 — Example of reads located in primer overlap between amplicons 2 and 3 for sample NA18870. A) All reads spanning the overlap region of amplicons 2 and 3. B) Reads remaining after eliminating those that start and end at the same position. The discarded reads are not identical to each other. (TIFF) [file pcbi.1002737.s002.tiff]

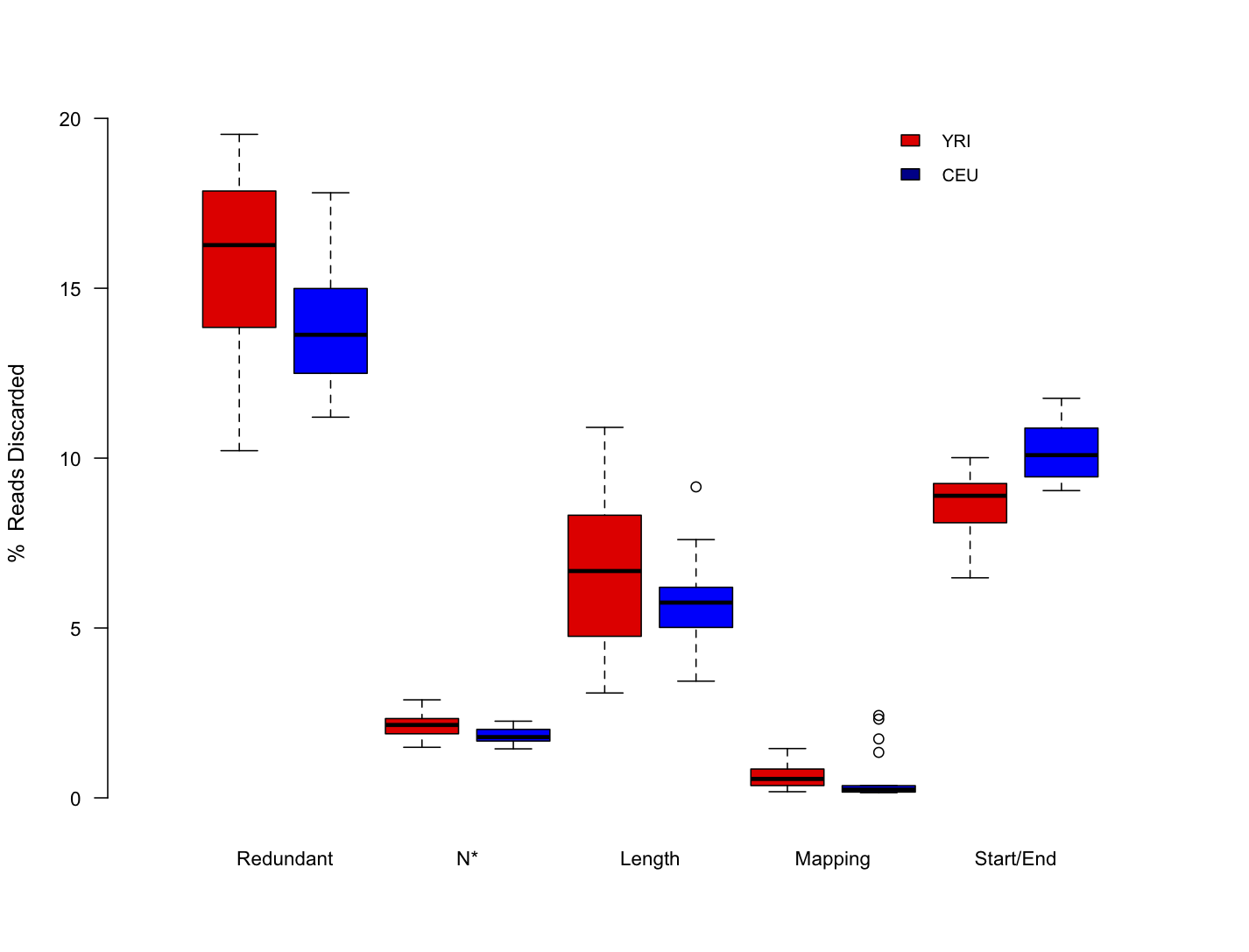

Supplement: Figure S3 — Quality control (QC) filters. Reads were filtered using five different criteria to retain only high quality reads by removing: 1. clonal reads; 2. reads containing at least one N (N does not indicate an ambiguous base but is defined as an instance when a nucleotide was not incorporated after three consecutive flows in a sequencing run); 3. reads longer than 300 or shorter than 200 nt based on the read length distribution observed across all samples; 4. reads mapping to multiple locations or not mapping to the mitochondrial genome; and, 5. reads of equal length starting and ending at the same positions but not identical to each other. On average 68% and 66% were preserved for CEU and YRI, respectively. (TIFF) [file pcbi.1002737.s003.tiff]

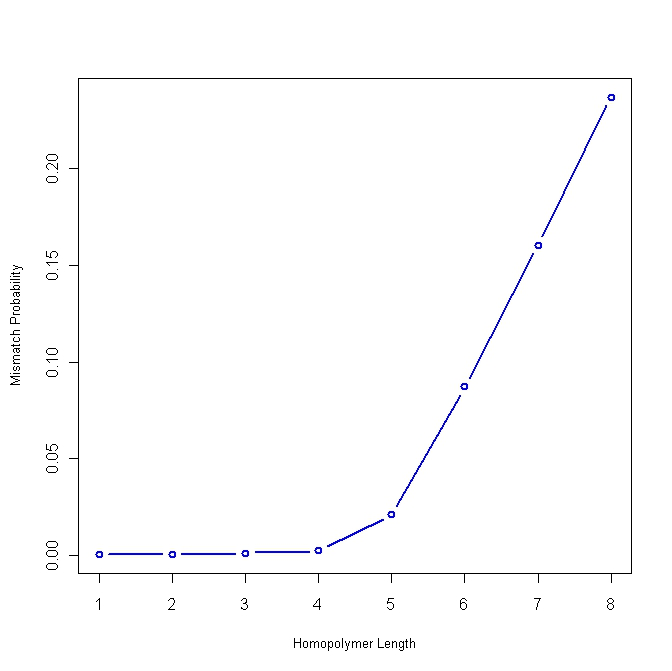

Supplement: Figure S4 — Sequence error as function of homopolymeric region. Homopolymer stretches were grouped by length and the total number of non-consensus bases within each stretch was divided by the total number of bases in that group. The probability of having a substitution or mismatch (majority are deletions) exponentially increases in homopolymer regions of length greater than 4 nt. Final positions in such regions were not called due to this intrinsic error in the 454 pyrosequencing base-calling algorithm along with the BLAST alignment. (TIFF) [file pcbi.1002737.s004.tiff]

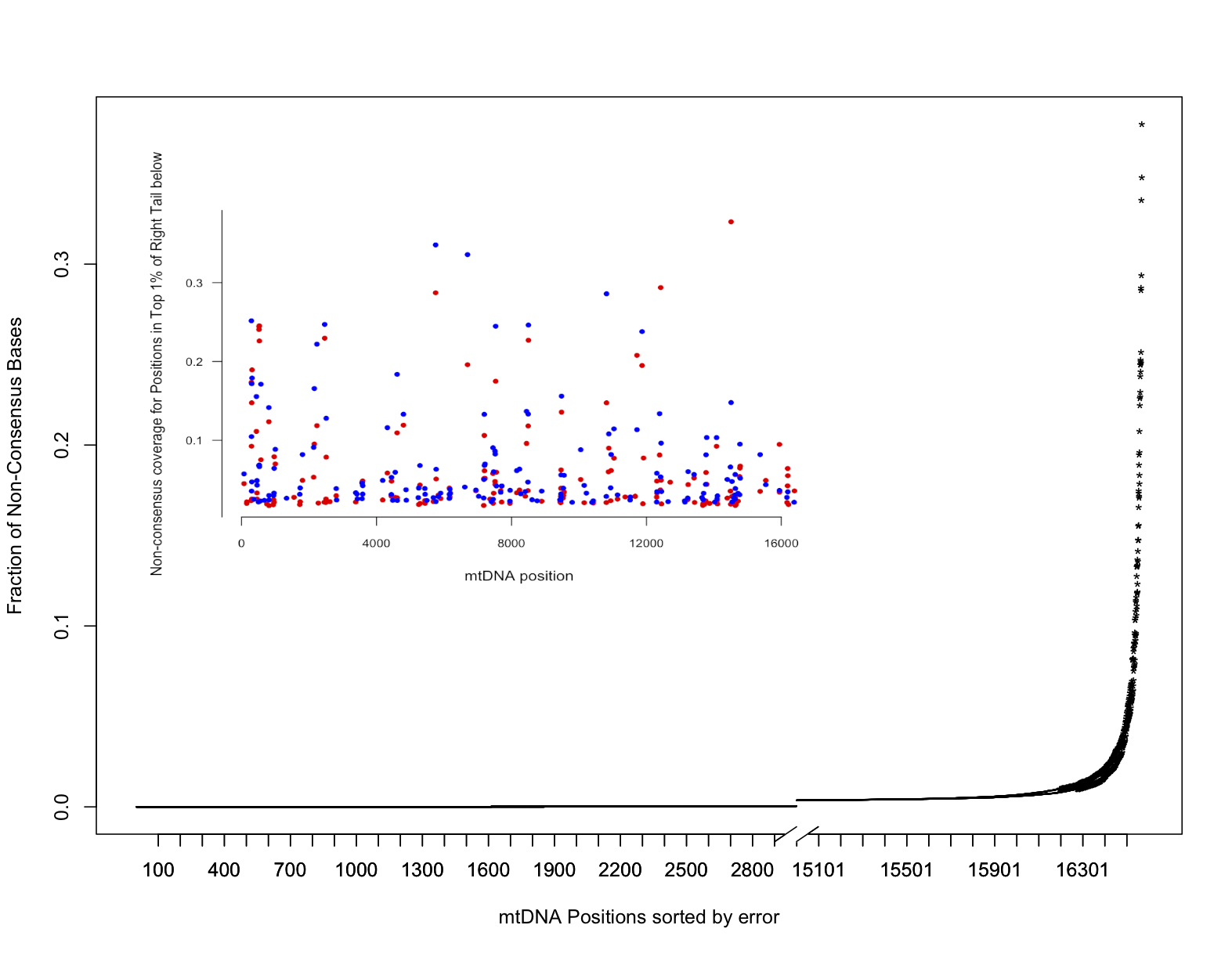

Supplement: Figure S5 — Fraction of total non-consensus bases. The fraction of total non-consensus bases was calculated by dividing the total number of non-consensus bases at a particular position by the total number of bases seen at that position. The results indicate that 99% of all positions have secondary coverage fraction less than 1.7×10^−2^, 2.1×10^−2^ for YRI, CEU respectively. (TIFF) [file pcbi.1002737.s005.tiff]

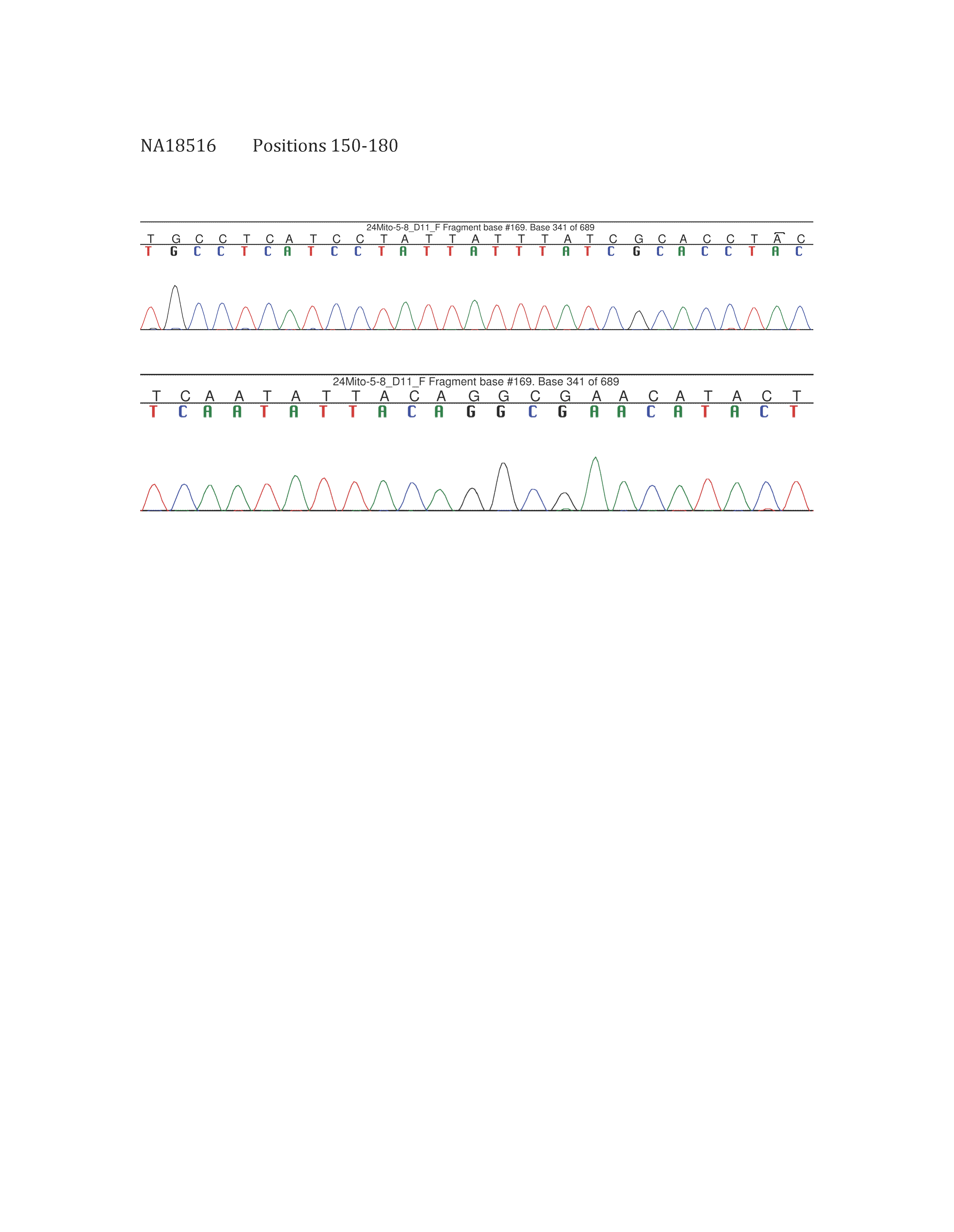

Supplement: Figure S6 — Sanger sequencing chromatograms for sample NA18516. The traces show the region containing 8 discrepancies between pyrosequencing and Sanger sequencing data. The pyrosequencing calls are also supported by 1000Genomes data. The whole region is covered by high quality bases. (TIFF) [file pcbi.1002737.s006.tiff]

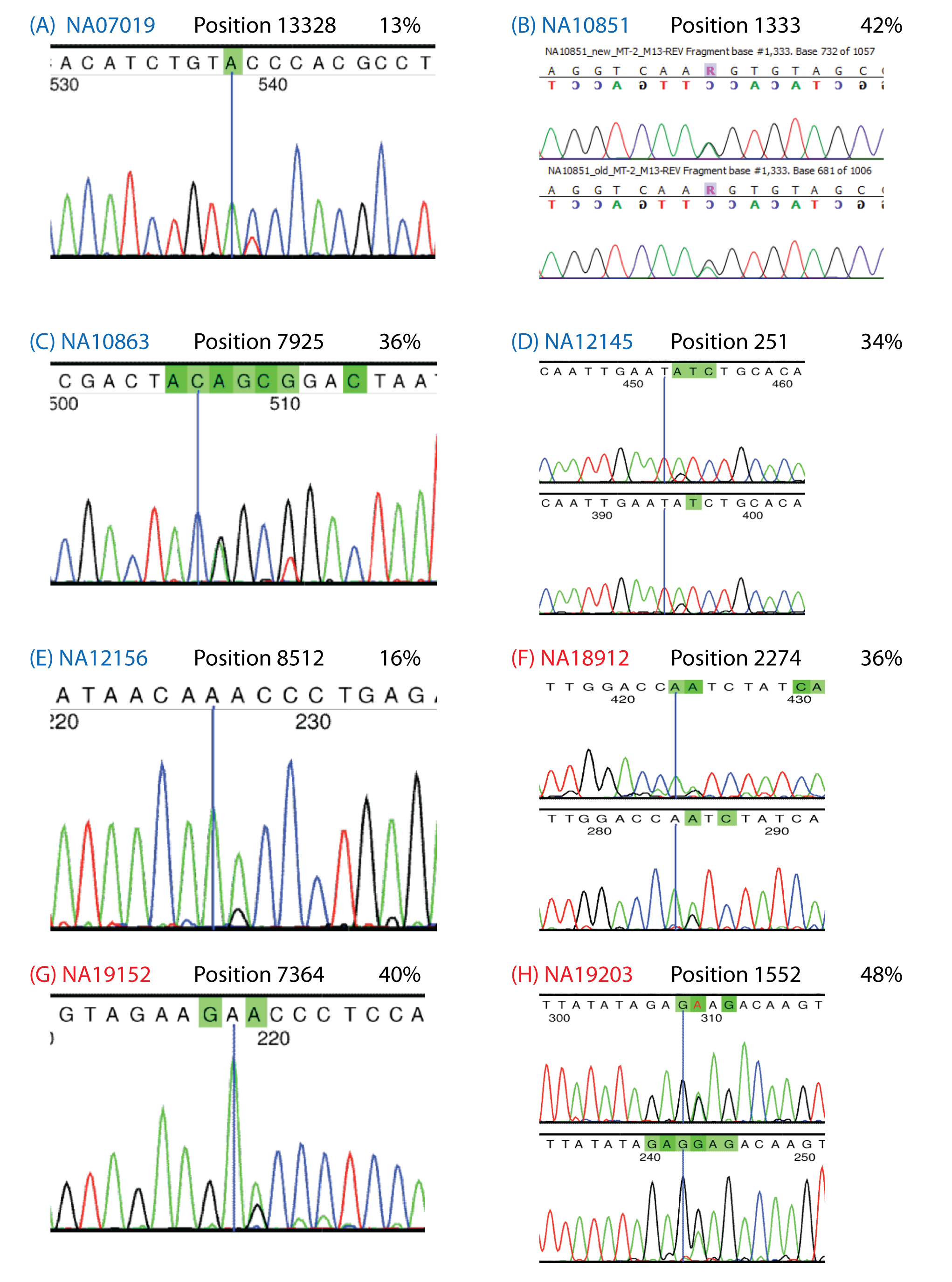

Supplement: Figure S7 — Sanger sequencing chromatograms showing heteroplasmic sites. Sanger sequence traces showing validation of both lower (10%–19%) and higher (>20%) level heteroplasmic sites. A) Bases around position 13328 for sample NA07019. Position 13328, next to the blue line, has two bases, namely a C and a T. The level of heteroplsmy for this site was calculated as 13% in our analysis. B) Position 1333 in sample NA10851 has two bases, G and A at almost 50%, close to the 42% in our analysis. C) Position 7925 in sample NA10863 next to the blue line, has two bases, G and A; calculated at 36% D) Position 251 in sample NA12145 next to blue line has two bases, G and A; calculated at 34%. E) Position 8512, next to blue line, shows a lower level heteroplasmic site (16%), A and G. F) Two traces covering the region around position 2274, show A and G heteroplasmy at that site, calculated as 36%. G) Position 7364 in sample NA19152 showing both A and G, calculated at 40%, and H) Position 1552 in sample NA19203 showing G and A, calculated at 48%. (TIFF) [file pcbi.1002737.s007.tiff]

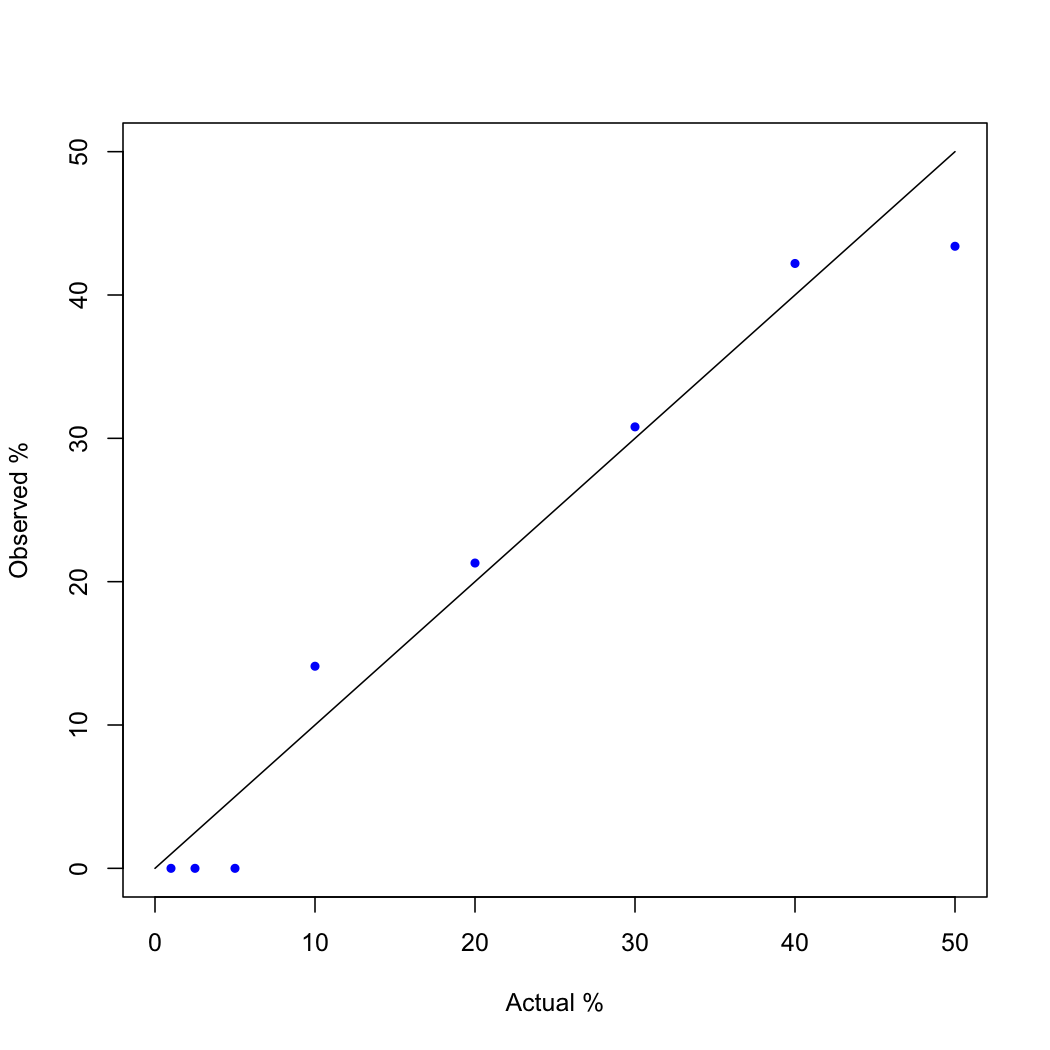

Supplement: Figure S8 — Accuracy of heteroplasmy detection. The Standard Material (SRM 2394) is designed to simulate different levels of heteroplasmy by mixing two 285 bp amplicons obtained from two different cell lines which differ by one nucleotide in the following mass percentages: 1%, 2.5%, 5%, 10%, 20%, 30%, 40%, and 50%. The graph shows the strong relationship between the true versus estimated mass percentage with coefficient of determination R2 = 0.96. (TIFF) [file pcbi.1002737.s008.tiff]

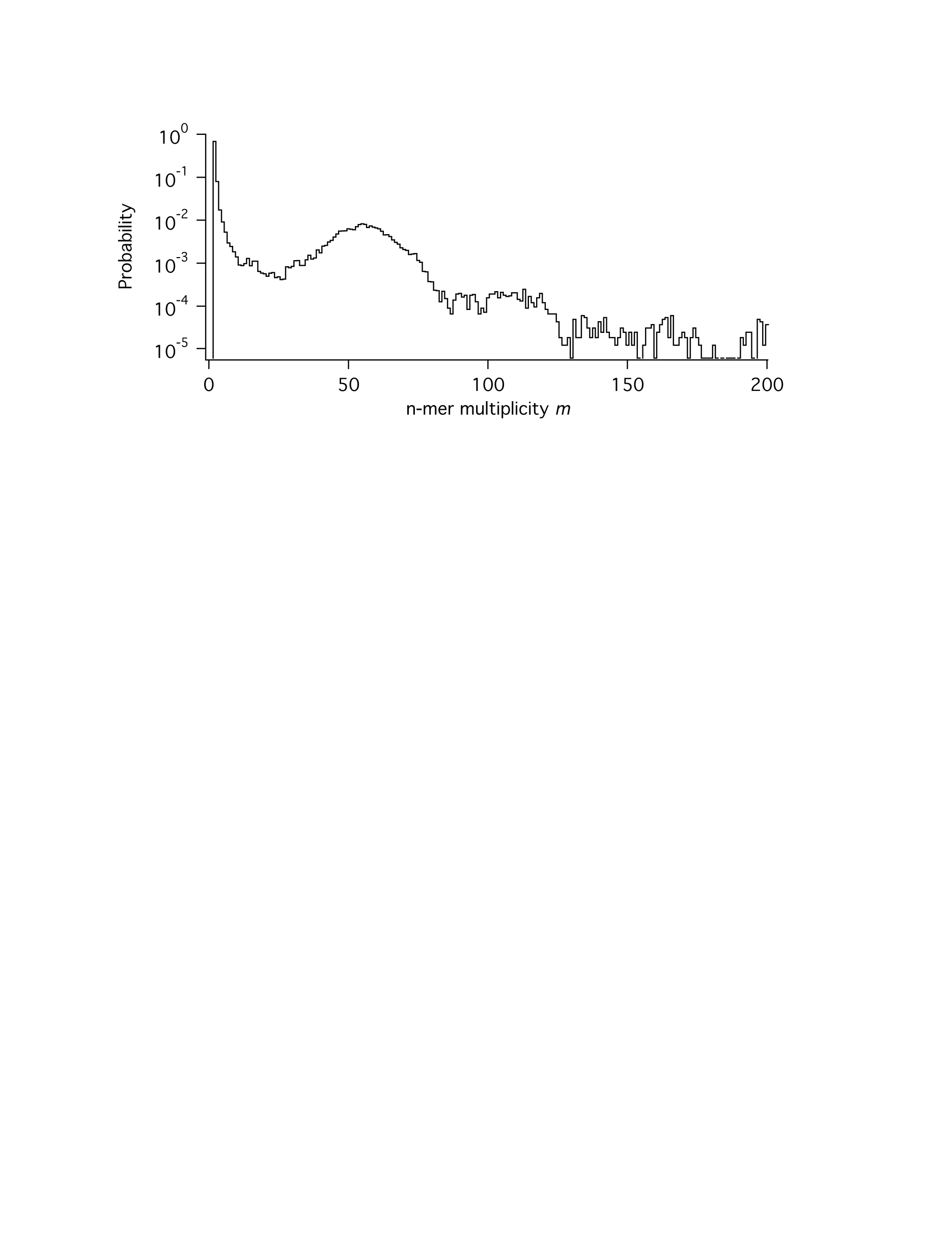

Supplement: Figure S9 — n-mer multiplicity. Frequency of occurrence (multiplicity) of n-mers for n = 27 obtained from 454 reads from a single HapMap sample (NA06993). (TIFF) [file pcbi.1002737.s009.tif]
