# Supplementary material for: Next-Generation Sequencing of Human Mitochondrial Reference Genomes Uncovers High Heteroplasmy Frequency
Source: PLoS Comput Biol. 2012 Oct 25;8(10):e1002737. doi: 10.1371/journal.pcbi.1002737 (PMC3486893; doi:10.1371/journal.pcbi.1002737)
Supplement: Table S1 — Heteroplasmic sites and validation status by sample. The table provides for each heteroplasmic site its position, the relevant bases, coverage, heteroplasmic frequency, validation status and whether it was also identified in 1000Genomes. A) Heteroplasmic sites found in YRI samples and B) CEU samples. (DOCX) [file pcbi.1002737.s010.docx]

## Table S1

## a.

| Sample ID | Position | Ref | Primary Base | Primary Base Coverage | Secondary Base | Secondary Base Coverage | % Hetero  plasmy | Status | 1000 Genomes |
| --- | --- | --- | --- | --- | --- | --- | --- | --- | --- |
| NA19092 | 150 | C | T | 117 | C | 43 | 27 |  |  |
| NA19203 | 1552 | G | G | 76 | A | 69 | 48 | validated |  |
| NA18500 | 2045 | A | G | 120 | A | 28 | 19 |  |  |
| NA18912 | 2274 | A | A | 81 | G | 46 | 36 | validated | X |
| NA18912 | 2838 | A | A | 85 | G | 24 | 22 |  |  |
| NA19171 | 4227 | A | A | 100 | G | 12 | 11 | validated |  |
| NA19152 | 7364 | A | A | 76 | G | 51 | 40 | validated |  |
| NA18870 | 10567 | T | T | 143 | C | 15 | 9 |  |  |
| NA19200 | 11371 | A | A | 82 | G | 61 | 43 | validated |  |
| NA19140 | 13602 | T | T | 60 | C | 11 | 15 | validated |  |
| NA19137 | 13722 | A | A | 87 | G | 31 | 26 | validated |  |
| NA18870 | 14352 | C | C | 63 | A | 13 | 17 |  | X |
| NA18516 | 14610 | T | T | 62 | C | 14 | 18 |  |  |

**B.**

| Sample ID | Position | Ref | Primary Base | Primary Base Coverage | Secondary base | Secondary Base Coverage | % Hetro  plasmy | Status | 1000  Genomes |
| --- | --- | --- | --- | --- | --- | --- | --- | --- | --- |
| NA12248 | 73 | A | G | 80 | A | 21 | 21 |  |  |
| NA12248 | 150 | C | T | 100 | C | 22 | 18 |  |  |
| NA12891 | 152 | T | C | 79 | T | 17 | 18 |  |  |
| NA12156 | 195 | T | C | 62 | T | 17 | 22 |  |  |
| NA10856 | 204 | T | C | 87 | T | 27 | 15 |  |  |
| NA12145 | 251 | G | A | 40 | G | 21 | 34 | validated |  |
| NA11882 | 673 | T | T | 90 | C | 45 | 33 |  |  |
| NA10851 | 1333 | G | G | 92 | A | 67 | 42 | validated* |  |
| NA12248 | 1721 | C | T | 115 | C | 14 | 11 |  |  |
| NA12891 | 2259 | C | T | 85 | C | 21 | 20 |  |  |
| NA12145 | 2407 | T | T | 77 | C | 15 | 16 |  |  |
| NA11831 | 2448 | G | G | 75 | A | 21 | 22 |  |  |
| NA12248 | 2706 | A | G | 118 | A | 16 | 12 |  |  |
| NA12891 | 2706 | A | A | 93 | G | 11 | 11 |  |  |
| NA12156 | 2927 | C | C | 116 | T | 19 | 14 | validated |  |
| NA12248 | 3010 | G | G | 124 | A | 19 | 13 |  |  |
| NA12248 | 3197 | T | C | 115 | T | 16 | 12 |  |  |
| NA12248 | 3212 | C | T | 112 | C | 17 | 13 |  |  |
| NA11882 | 3591 | G | G | 79 | A | 78 | 50 | validated |  |
| NA12248 | 4067 | T | T | 103 | C | 17 | 14 |  |  |
| Sample ID | Position | Ref | Primary Base | Primary Base Coverage | Secondary base | Secondary Base Coverage | % Hetro  plasmy | Status | 1000  Genomes |
| NA12760 | 4502 | T | T | 104 | C | 13 | 11 |  |  |
| NA12248 | 4732 | A | G | 89 | A | 11 | 11 |  |  |
| NA12891 | 4745 | A | G | 81 | A | 13 | 14 |  |  |
| NA12248 | 5460 | G | G | 89 | A | 17 | 16 |  |  |
| NA12891 | 6266 | A | A | 50 | G | 21 | 30 |  |  |
| NA12891 | 6755 | G | G | 92 | A | 25 | 21 |  |  |
| NA12145 | 7028 | C | T | 64 | C | 33 | 34 |  |  |
| NA12891 | 7028 | C | C | 95 | T | 20 | 17 |  |  |
| NA12891 | 7337 | G | A | 105 | G | 30 | 22 |  |  |
| NA10863 | 7524 | T | T | 81 | C | 25 | 24 |  |  |
| NA10863 | 7925 | G | G | 82 | A | 46 | 36 | validated |  |
| NA12156 | 8512 | A | A | 114 | G | 21 | 16 | validated |  |
| NA12145 | 9055 | G | A | 39 | G | 35 | 47 |  |  |
| NA12146 | 9095 | T | C | 45 | T | 43 | 49 | validated |  |
| NA12156 | 9501 | T | T | 96 | C | 16 | 14 |  |  |
| NA12145 | 9698 | T | C | 63 | T | 37 | 37 |  |  |
| NA12145 | 10398 | A | G | 58 | A | 39 | 40 |  |  |
| NA12145 | 10550 | A | G | 57 | A | 47 | 45 |  |  |
| NA12145 | 11299 | T | C | 44 | T | 26 | 37 |  |  |
| NA12145 | 11467 | A | A | 44 | G | 39 | 47 |  |  |
| NA12144 | 12303 | C | C | 134 | T | 25 | 16 |  | X |
| NA12248 | 12372 | G | A | 80 | G | 12 | 13 |  |  |
| NA11882 | 12642 | A | A | 118 | G | 53 | 31 |  |  |
| NA07019 | 13328 | C | C | 70 | T | 10 | 13 | validated |  |
| NA10854 | 13466 | G | G | 152 | A | 26 | 15 |  |  |
| NA12760 | 13558 | G | A | 73 | G | 36 | 33 |  |  |
| NA12248 | 13617 | T | C | 89 | T | 16 | 15 |  |  |
| NA12248 | 13637 | A | G | 84 | A | 15 | 15 |  |  |
| NA12891 | 14872 | C | T | 107 | C | 13 | 11 |  |  |
| NA12248 | 14902 | C | C | 85 | T | 10 | 11 |  |  |
| NA12248 | 14956 | T | C | 91 | T | 11 | 11 | validated |  |
| NA11995 | 15663 | T | T | 191 | C | 23 | 11 |  |  |
| NA12248 | 16172 | T | T | 60 | C | 10 | 14 |  |  |
| NA12248 | 16192 | C | C | 57 | T | 8 | 12 |  |  |
| NA12248 | 16270 | C | T | 53 | C | 10 | 16 |  |  |
| NA12145 | 16284 | A | A | 55 | G | 8 | 13 | validated |  |
| NA12248 | 16312 | A | A | 59 | G | 11 | 16 |  |  |
| NA12248 | 16519 | T | T | 90 | C | 15 | 14 |  |  |
| *validated by TaqMan as well | | | |  |  |  |  |  |  |
