# Supplementary material for: Next-Generation Sequencing of Human Mitochondrial Reference Genomes Uncovers High Heteroplasmy Frequency
Source: PLoS Comput Biol. 2012 Oct 25;8(10):e1002737. doi: 10.1371/journal.pcbi.1002737 (PMC3486893; doi:10.1371/journal.pcbi.1002737)
Supplement: Table S2 — Positional distribution of a particular heteroplasmic site along a read. The symbol c( ) is used to count the number of times the primary base at any position is found at the beginning, middle or end of a read, and d( ) is used in the same way for counts of the secondary base at that position. A) Two strong heteroplasmic candidate sites showing a uniform positional distribution. B) Three artifactual heteroplasmic candidate sites displaying a biased distribution along the read. These sites are all contained in primer sequences. (DOCX) [file pcbi.1002737.s011.docx]

**Table S2**

**A.**

| Sample | Position | c(head) | c(mid) | c(tail) | d(head) | d(mid) | d(tail) |
| --- | --- | --- | --- | --- | --- | --- | --- |
| NA10851_1 | 1333 | 28 | 41 | 26 | 20 | 23 | 30 |
| NA10851_2 | 1333 | 6 | 9 | 10 | 9 | 6 | 7 |
| NA10856_1 | 204 | 7 | 14 | 10 | 30 | 37 | 29 |
| NA10856_2 | 204 | 6 | 6 | 2 | 27 | 27 | 18 |

**B.**

| Sample | Position | c(head) | c(mid) | c(tail) | d(head) | d(mid) | d(tail) |
| --- | --- | --- | --- | --- | --- | --- | --- |
| NA18912 | 11719 | 252 | 0 | 40 | 13 | 9 | 205 |
| NA18912 | 11914 | 223 | 0 | 72 | 8 | 12 | 201 |
| NA18912 | 16390 | 121 | 1 | 9 | 25 | 35 | 28 |
